# Umami taste perception and preferences of the domestic cat (*Felis catus*), an obligate carnivore

**DOI:** 10.1093/chemse/bjad026

**Published:** 2023-08-08

**Authors:** Scott J McGrane, Matthew Gibbs, Carlos Hernangomez de Alvaro, Nicola Dunlop, Marcel Winnig, Boris Klebansky, Daniel Waller

**Affiliations:** Waltham Petcare Science Institute, Freeby Lane, Waltham-on-the-Wolds, Melton Mowbray, Leicestershire LE14 4RT, United Kingdom; Waltham Petcare Science Institute, Freeby Lane, Waltham-on-the-Wolds, Melton Mowbray, Leicestershire LE14 4RT, United Kingdom; Waltham Petcare Science Institute, Freeby Lane, Waltham-on-the-Wolds, Melton Mowbray, Leicestershire LE14 4RT, United Kingdom; Waltham Petcare Science Institute, Freeby Lane, Waltham-on-the-Wolds, Melton Mowbray, Leicestershire LE14 4RT, United Kingdom; AXXAM GmbH, Byk-Gulden Str.2, 78467 Constance, Germany; BioPredict, Inc., 4 Adele Avenue, Demarest, NJ 07627, United States; Waltham Petcare Science Institute, Freeby Lane, Waltham-on-the-Wolds, Melton Mowbray, Leicestershire LE14 4RT, United Kingdom

**Keywords:** pet, palatability, receptor, in silico, in vitro, in vivo

## Abstract

The domestic cat (*Felis catus*) is an obligate carnivore, and as such has a meat-based diet. Several studies on the taste perception of cats have been reported, indicating that their sense of taste has evolved based on their carnivorous diet. Here, we propose that umami (mediated by Tas1r1-Tas1r3) is the main appetitive taste modality for the domestic cat by characterizing the umami taste of a range of nucleotides, amino acids, and their mixtures for cats obtained using complementary methods. We show for the first time that cats express *Tas1r1* in taste papillae. The cat umami receptor responds to a range of nucleotides as agonists, with the purine nucleotides having the highest activity. Their umami receptor does not respond to any amino acids alone; however, 11 l-amino acids with a range of chemical characteristics act as enhancers in combination with a nucleotide. l-Glutamic acid and l-Aspartic acid are not active as either agonists or enhancers of the cat umami receptor due to changes in key binding residues at positions 170 and 302. Overall, cats have an appetitive behavioral response for nucleotides, l-amino acids, and their mixtures. We postulate that the renowned palatability of tuna for cats may be due, at least in part, to its specific combination of high levels of inosine monophosphate and free l-Histidine that produces a strong synergistic umami taste enhancement. These results demonstrate the critical role that the umami receptor plays in enabling cats to detect key taste compounds present in meat.

## Introduction

The domestic cat (*Felis catus*) is an “obligate” or “true” carnivore, meaning that it eats a meat-based diet and requires nutrients only found in meat (Bradshaw 1991, 2006). In a series of papers published by Boudreau and colleagues in the 1970s, electrophysiology recordings using neurons in the geniculate ganglion (facial nerve) of cats established that they were responsive to meats and meat-based compounds (Boudreau et al. 1971, 1975; Boudreau and Alev 1973; Boudreau 1977; Boudreau and Nelson 1977). Furthermore, it has been shown more recently that cats will select diets with high protein and fat contents, but not diets with high carbohydrate content, when given a choice of foods with different macronutrient profiles (Hewson-Hughes et al. 2011; Salaun et al. 2017).

Since the discovery of the taste receptors, several studies investigating the different taste modalities of cats have been reported, indicating that their sense of taste has evolved based on their diet of meat and is in many cases different from that of humans. It is well known that cats are indifferent to sugars and sweeteners (Carpenter 1956; Bartoshuk et al. 1975; Beauchamp et al. 1977), which is due to their sweet taste receptor gene (*Tas1r2*) being pseudogenized (Li et al. 2005). One explanation is that cats lost their ability to taste sugar as they do not commonly encounter it in their strictly carnivorous diet. Cats have less bitter taste receptors than humans, and some differences in the receptive ranges compared to humans have also been reported (Lei et al. 2015; Sandau et al. 2015). Indeed, it has been proposed that the occurrence of bitter and toxic foods is lower for carnivores compared to herbivores or omnivores (Glendinning 1994). Salt (NaCl) taste receptors have not been studied specifically for cats. However, it has been reported that cats don’t respond to lower salt concentrations (≤0.05 M) that cause responses in other non-carnivorous species (Boudreau et al. 1985) and that there is an absence of salt preference or appetite in sodium-replete or depleted kittens (Yu et al. 1997). A potential explanation of the low sensitivity of cats to NaCl may be due to the high sodium content of meat (Bradshaw 1991). Recently, we compared the kokumi taste perception of the domestic cat to humans and identified broad similarities in ligand specificity, but differences in taste sensitivity (Laffitte et al. 2021).

The term “umami” was first coined by K. Ikeda in 1908, who discovered this important taste modality, which is now one of the 5 basic taste sensations and is responsible for savory or meaty taste (Yamaguchi and Ninomiya 2000). Umami taste perception in mammals is primarily mediated by the Tas1r1-Tas1r3 heterodimer. Tas1r1 and Tas1r3 are both class C G protein-coupled receptors (GPCRs) (Zhao et al. 2003; Ahmad and Dalziel 2020). The unique motif in class C GPCRs is a large extracellular N-terminus that is often referred to as the Venus fly trap (VFT) domain, which is tethered by a cysteine-rich region to a seven-transmembrane (7TM) domain (Chun et al. 2012). The response profile of the umami receptor has now been studied in several species, including humans (Li et al. 2002), mice (Nelson et al. 2002), and pigs (Roura et al. 2011). A range of activities have been identified for Tas1r1-Tas1r3, from narrowly tuned to broadly tuned; the human umami receptor responds only to the acidic amino acids l-Glutamic acid (and the sodium salt, monosodium glutamate [MSG]) and to a lesser extent l-Aspartic acid (Li et al. 2002), while the mouse umami receptor responds to multiple amino acids with different chemical characteristics, but not the acidic amino acids (Nelson et al. 2002).

However, there has only been limited research published on cat umami taste perception to-date. We have previously reported studies on the response profile of the cat umami receptor using in silico modeling (McGrane et al. 2013) and also a novel approach for functional characterization using only the Tas1r1 VFT domain and a small subset of amino acids (Belloir et al. 2017). In the modeling work, we confirmed that 2 key amino acid substitutions (ALA170GLU and ALA302ASP), which were previously identified by comparing the human and mouse receptors are present in the cat Tas1r1 amino acid binding site that significantly changed its binding properties, resulting in l-Glutamic acid and l-Aspartic acid being inactive. In the functional characterization work with the VFT of the cat Tas1r1, we used intrinsic tryptophan fluorescence to measure conformational changes in cat Tas1r1 VFT due to ligand binding for 5 amino acids with different chemical properties. Four of these amino acids (l-Alanine, l-Arginine, l-Histidine, and l-Isoleucine) were identified as active, while l-Cysteine was identified as being inactive. The nucleotide Inosine 5ʹ-monophosphate (IMP) was shown to potentiate l-amino acid binding. Using functional expression of cat Tas1r1-Tas1r3, it was recently shown that the purine nucleotides IMP and Guanosine 5ʹ-monophosphate (GMP) strongly activated the receptor, but l-Glutamic acid was inactive alone (Toda et al. 2021).

Here, we report results on the umami taste of nucleotides, amino acids, and mixtures for the domestic cat obtained using a combination of in silico, in vitro, and in vivo methods. We confirm that nucleotides are strong agonists of the cat Tas1r1-Tas1r3 umami receptor and demonstrate that amino acids are not active alone; however, a subset of l-amino acids act as enhancers in combination with a nucleotide. Indeed, these compounds and their mixtures are highly appetitive for cats, which is consistent with their evolution as obligate carnivores.

## Materials and methods

The standard gene and gene product nomenclature has been used here for human and mouse: *TAS1R1/3* for human genes and TAS1R1/3 for human gene products, and *Tas1r1/3* for mouse genes and Tas1r1/3 for mouse gene products. We have also used the same nomenclature for cat (and any other species mentioned) here as is used for mouse, since this has been used previously (Li et al. 2005).

### Expression of Tas1r1 in cat taste papillae

Only expression of cat *Tas1r3*, not *Tas1r1*, has been reported previously (Li et al. 2005). Hence, to confirm the expression of *Tas1r1* in the taste papillae of the cat, RT-PCR was conducted for *Tas1r1* with Glyceraldehyde 3-phosphate dehydrogenase (*Gapdh*) used as a control for non-taste tissue. Tissue was provided through collaboration with the University of Veterinary Medicine in Hannover, Germany. Tissue samples from a 6-year-old male cat were taken after euthanasia due to clinical reasons. The cat was client-owned, and permission to retrieve the samples was obtained prior to this work. Fungiform papillae from across the anterior section of the tongue surface were used for total RNA extraction along with a section tongue epithelium containing no visible taste papillae. RNA was extracted using the RNeasy Plus Mini Kit (Qiagen, Germany) according to the manufacturer’s instructions. cDNA synthesis was performed with the Superscript III First Strand cDNA Synthesis Kit (Thermo Fisher Scientific, UK) with random hexamer-based priming. The intron spanning primers used were as follows, *Tas1r1*, 5ʹ-CGGGAGCTTCCTCAACAAGA-3ʹ (forward) and 5ʹ-AGATGGTCTCGTGCCAAGTC-3ʹ (reverse); *GAPDH* 5ʹ-GTGAAGGTCGGAGTCAACGG-3ʹ (forward); and 5ʹ-ACCATAAGGTCCACCACCCG-3ʹ (reverse). For PCR, 25 µL reactions were prepared with JumpStart Taq ReadyMix (Sigma-Aldrich, UK) according to the manufacturer’s instructions. PCR was performed with an initial denaturation step of 94 °C for 2 min, followed by 35 cycles of denaturation at 94 °C for 30 s, annealing at 55 °C (*Tas1r1*) or 59 °C (*GAPDH*) for 30 s, and extension at 74 °C for 2 min. A final elongation step at 72 °C for 5 min was performed before the reactions were held at 4 °C until they were stored at −20 °C. Reaction products were run on a 1% agarose gel with post-staining in GelRed (Biotium, USA).

### Generation of cat Tas1r1 and Tas1r3 reference sequences

To confirm the sequence of cat *Tas1r1* and *Tas1r3* for use in silico and in vitro, DNA from 15 domestic shorthair cats was collected using cheek swabs. All exons of the two genes were re-sequenced using Sanger sequencing. Briefly, PCR products were generated using flanking primers, 10 ng DNA and a PCR Master Mix (Kapa Biosystems). PCR products were purified using solid-phase reversible immobilization chemistry (AMPure - Beckman Coulter Genomics) followed by dye-terminator fluorescent sequencing. Sequencing fragments were detected via capillary electrophoresis using ABI Prism 3730xl DNA analyzers (Applied Biosystems, Foster City, USA). A further 547 cats of varying breeds were sequenced to check the cat *Tas1r1* and *Tas1r3* sequences for commonly occurring variants (see Supplementary Table 1 for a breakdown of all cat breeds used to derive the reference sequences). All variants were assessed, and the final sequences used contained the most commonly occurring versions.

### Sequence alignments and phylogenetic tree

A selection of Tas1r1 mammalian protein sequences was retrieved from the Ensembl genome browser (www.ensembl.org). Sequences were selected to cover mammals with different dietary habits, including carnivores, omnivores, and herbivores. Sequences were aligned using CLC Genomics Workbench (v12) using the default alignment parameters. The resulting alignment file was used with iTOL (Letunic and Bork 2016) to generate the phylogenetic tree.

### Modeling of amino acid and nucleotide binding to cat and human Tas1r1 VFT

The model of the human TAS1R1 structure is taken from the AlphaFold Protein Structure Database (www.alphafold.ebi.ac.uk) (Jumper et al. 2021). This structure was used as the template for our homology model of the cat Tas1r1 VFT constructed using the Modeler software, which includes both the sequence alignments and the building of the homology model (Discovery Studio- BIOVIA, Dassault Systèmes) (Webb and Sali 2016). Cat Tas1r1 has ~80% sequence identity to human TAS1R1 with some important mutations within the active site of the VFT domain. The amino acids were docked deep within the hinge region of the human and cat Tas1r1 VFT with the amino acid groups positioned similar to the amino acid groups in other class C GPCR structures (Zhang et al. 2008) using the program BioDock (Pedretti et al. 2002) (see Fig. 2B for l-Glutamic acid and l-Alanine docked into the human and cat models of the Tas1r1 VFT, respectively). IMP was docked into the human and cat Tas1r1 VFT according to the modeing results by one of the authors in (Zhang et al. 2008) for human TAS1R1 also using the program BioDock (Pedretti et al. 2002; Fig. 2B). The resulting complexes were subjected to energy minimization using the GROMACS software package (Berendsen et al. 1995). Finally, the results were ranked manually by energetic criteria that include hydrogen bonding, charged, and hydrophobic interactions using CHARMM force fields (MacKerell et al. 1998).

### Functional expression of cat Tas1r1-Tas1r3

#### Assay development

Development of the cat Tas1r1-Tas1r3 assay was adapted from a previously published method (Li et al. 2004). All experiments were performed using HEK293T cells stably transfected with the G-protein chimera mGα15i1 comprised of the first 369 amino acids of mGα15 and the last 5 amino acids (DCGLF) of mGαi1 (see Supplementary Fig. 1 for the mGα15i1 amino acid sequence). Cat *Tas1r1* and cat *Tas1r3* were also stably transfected, but the GeneSwitch system (Invitrogen) was used, resulting in expression which had to be induced by adding 0.1 nM mifepristone to the cells 48 h prior to the experiment. The coding sequences of the *Felis catus Tas1r1* and *Tas1r3* genes were synthesized by GeneArt (Thermo Fisher Scientific). The cat coding sequences were excised from the GeneArt construct and sub-cloned into the pGene expression vector for inducible expression. Expression constructs were transfected into the HEK293T cell line using Lipofectamine 2000 according to the manufacturer’s recommendations. A stable cell line with robust responses to l-Alanine and IMP was selected by 2 rounds of limiting dilution. Cells not exposed to mifepristone were unresponsive. The cell line was maintained in Dulbecco’s MEM High-Glucose supplemented with Glutamax-I (Gibco) and with 10% dialyzed fetal bovine serum, 1% Penicillin/Streptomycin, 100 µg/mL G418, 100 µg/mL Hygromycin, 10 µg/mL Zeocin, 2.5 µg/mL Blasticidin, and 0.5 µg/mL Puromycin. Standard propagation conditions consisted of seeding with cells at 80% confluence from a 10Ø petri dish at a ratio of 1:10 twice a week.

#### Preparation of umami compounds

l-Alanine and IMP were used as reference compounds, as they robustly activate the cat umami receptor in combination. These and other test compounds were stored at −20 °C and freshly prepared in the assay buffer on the day of the experiments. The assay buffer was comprised of 5 mM KCl, 130 mM NaCl, 2 mM CaCl_2_, 5 mM NaHCO_3_, 1 mM MgCl_2_, and 20 mM HEPES, with pH adjusted to 7.4 (Tyrode’s buffer). All nucleotides and amino acids were obtained from Sigma-Aldrich (St. Louis, MO, USA) and were of the highest available purity. For the functional expression work, the solubility of amino acids was improved by sonication. l-Tryptophan was insoluble at 60 mM. l-Tyrosine showed precipitation at 30 mM and 60 mM, thus the indicated concentrations for l-Tyrosine should be considered as approximate.

#### Calcium mobilization assay

The calcium mobilization experiments were performed using a FLIPR Tetra (Molecular Devices). Cells were seeded in black 384-well polystyrene assay plates with a clear bottom and a Poly-d-Lysine coating (Matrix CPL-4332) 72 h prior to the experiment at 15,000 cells/well. Twenty-four hours later, 0.1 nM mifepristone was added to each well to induce expression of cat Tas1r1 and cat Tas1r3. Cells that did not receive the mifepristone treatment (uninduced) were generally unresponsive to umami taste stimuli. Forty-eight hours after induction with mifepristone, the medium was removed from the cells and 20 μL loading buffer (Tyrode’s buffer + 2 μM Fluo4 AM + 100 μM probenecid) was added, and the plate was incubated for 40 min at 37 °C and then transferred for another 20 min at room temperature. The cells were then washed two times with Tyrode’s buffer using an automated plate washer (Biotek) and incubated for 20 min at room temperature in the dark. The cells were then washed a further two times before being transferred to the FLIPR Tetra. In the experiments with nucleotides in combination with l-Alanine, the concentration of l-Alanine used was 20 mM, and in the experiments with amino acids in combination with IMP, the concentration of IMP used was 0.2 mM.

#### Data analysis

Concentration–response curves, each with 8 concentration points, were established by plotting signal amplitudes versus agonist concentration. The half maximal effective concentrations (EC_50_) were identified by nonlinear regression using a variable slope model with the equation Y = Bottom + (X^Hillslope) × (Top-Bottom)/(X^HillSlope + EC50^HillSlope), where Y is the response, and X is the agonist concentration, Top and Bottom are the plateaus in the same units as Y and HillSlope is the Slope factor or Hill slope. All calculations and plots were done using GraphPad Prism 8 (GraphPad Software, CA). Several tested conditions elicited a cellular response only at 1 or 2 concentrations and did not reach a plateau, preventing us from using the above equation to calculate an EC_50_ value for these ligands. In these cases, the EC_50_ was noted as being higher than the maximum concentration used in the assay.

### Taste choice tests with taste compounds using cat water panel testing

#### Animals and housing

The minimum panel size was *n* = 14, but only for one study with 80 mM MSG; in all other cases, the minimum panel size was *n* = 21. The maximum panel size was *n* = 24. In some cases, multiple studies were run on the same compound, which is why the *n*-value is higher. For those cases, we have also reported the number of unique cats, as some cats were present in more than one study. All cats on the panel had previously been habituated and screened for suitability to perform this type of study. The cats had an age of 1.77–11.67 years, with the proportion of males varying between 0.35 and 0.75 (accordingly, the proportion of females varied between 0.25 and 0.65) during the studies. The behaviorally enriched housing conditions of the cats has been described previously (Hewson-Hughes et al. 2011; Laffitte et al. 2021). The cats were re-homed once they completed their tenure at the Waltham Petcare Science Institute, as per standard Waltham practice.

#### Taste stimuli

Solutions were made with deionized water (Purite, UK). All taste-active compounds were food-grade or of the highest available purity. In some cases, salts were purchased instead of the pure compound due to solubility, stability, or availability. For all the solutions, the pH range spanned approximately 5–8.

#### Experimental protocol

Choice tests for the taste compound solutions at specific concentrations were run over an 18-h period using a 2-bottle testing apparatus. Full details of the methodology are reported in Laffitte et al. (2021). All animal studies conformed with the Mars Animal Research Policy (www.Mars.com) and were approved by the Waltham Animal Welfare and Ethical Review Body. They also followed the 3Rs approach to experimentation with animals in scientific research (Robinson 2005) and were conducted in accordance with local guidelines and regulations. The reporting of the animal studies conforms to the ARRIVE guidelines (Kilkenny et al. 2010).

#### Data analysis

Using a bespoke statistical analysis toolkit, based on R software (R Foundation, Vienna, Austria), a mixed model analysis was performed on the difference in intake (g) between water solutions, including cat (random) as a factor where appropriate. This was used to test the mean intake difference versus 0, i.e. no difference, at a 5% significance level. The mean difference in intake was reported with a 95% confidence interval. Any data errors (e.g. following manual error such as solution spillage, overflow) were removed from the final data set for analysis.

## Results

### Tas1r1 and Tas1r3 are expressed in the cat fungiform taste papillae

**Fig. 1.**
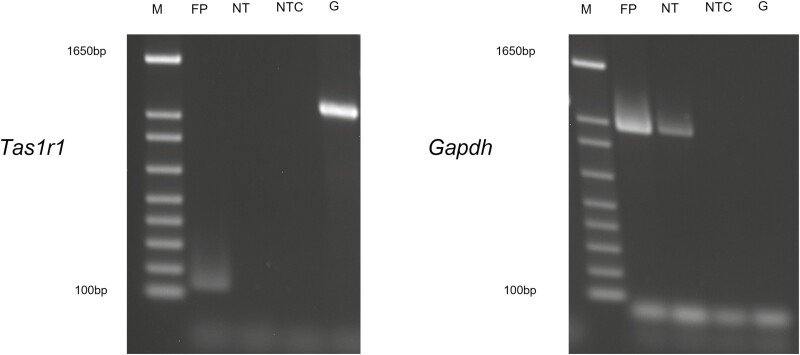
Tas1r1 is expressed in cat fungiform taste papillae. RT-PCR for cat fungiform papillae (FP), nontaste epithelial tissue (NT), no-template controls (NTC) and genomic DNA (G). Gapdh was used as a positive control. Expression of Tas1r1 was only observed in the FP. The genomic DNA amplicon for Gapdh was too large to amplify in this PCR. M is the molecular size marker.

We used biopsies of cat fungiform papillae to perform an RT-PCR analysis to confirm that cats express *Tas1r1* in their taste tissue (Fig. 1). Expression of *Tas1r1* was identified, with no expression being observed in epithelial tongue tissue without visible papillae. *GAPDH* was used as a positive control in both tissue samples. Reactions with no template cDNA showed no amplification products. Control reactions with genomic DNA as template showed a high molecular weight product with *Tas1r1* primers. The genomic DNA control for *GAPDH* did not show a product, as the expected size of this amplicon would be 3927bp, and hence the extension time used here would not be sufficient (see Supplementary Fig. 2 for full-length gels). Expression of *Tas1r3* in cats was reported previously (Li et al. 2005).

### Cat Tas1r1 and Tas1r3 reference sequences

Reference sequences for cat Tas1r1 (see Supplementary Fig. 3 for full sequence) and Tas1r3 (see Supplementary Fig. 4 for full sequence) were generated by sequencing 562 cats. The sequence of cat *Tas1r1* we generated matched the sequence found on the Ensembl database (www.ensembl.org, Felis_catus_9.0) at the time of writing, with only one synonymous variation present. In the case of *Tas1r3*, other synonymous variations occurred along with a single coding variant. When compared with the sequence in the Ensembl database, our sequence contained a TRP255ARG variation. We found the ARG variant to be the major allele in cat samples, with an allele frequency of 0.994. The cat Tas1r1 and Tas1r3 sequences were used to generate the phylogenetic tree, the homology model for the cat Tas1r1 and the cat in vitro umami (Tas1r1-Tas1r3) assay used in this study.

### The cat Tas1r1 VFT binding site and activity

**Fig. 2.**
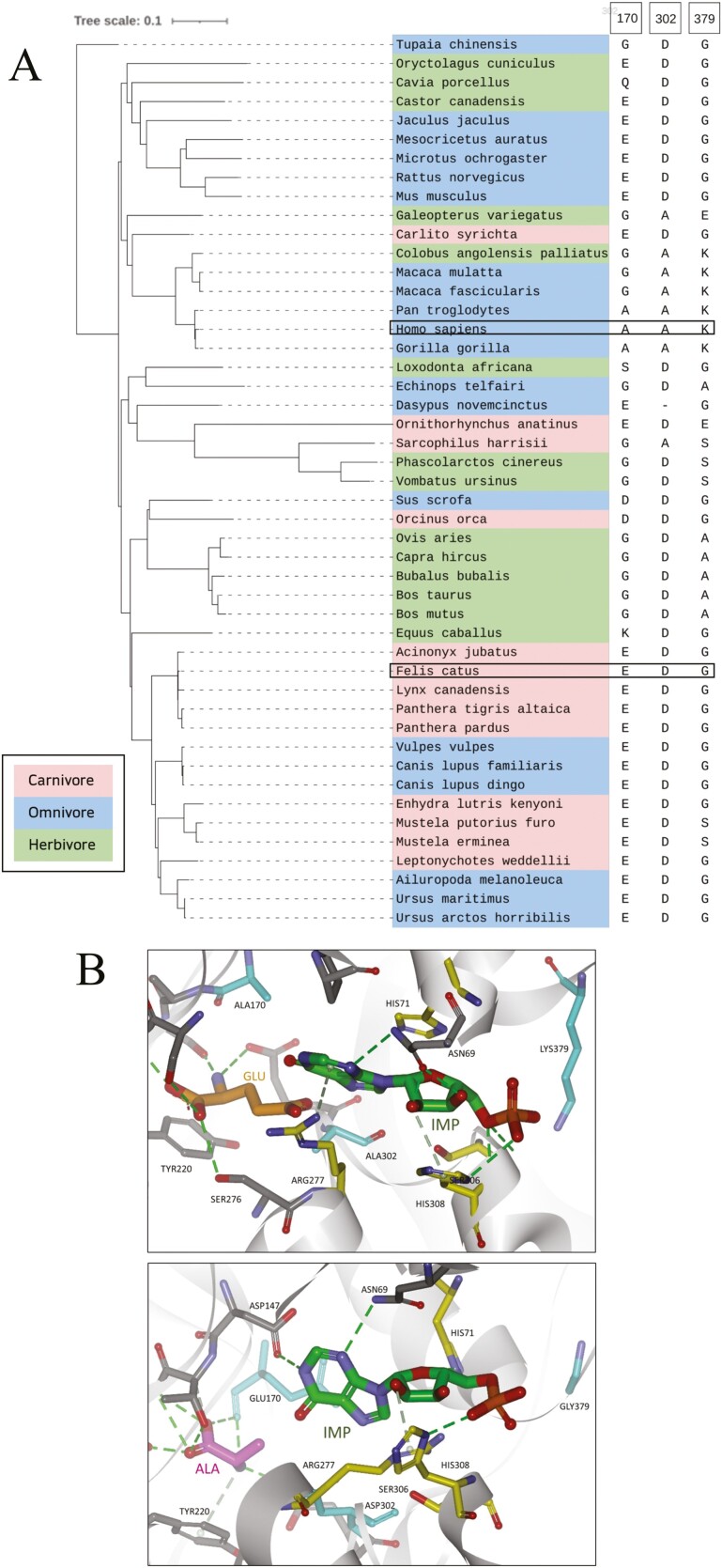
(A) Tas1r1 phylogenetic tree categorized with carnivores (red), omnivores (blue), and herbivores (green) highlighted and summary of key amino acids at positions 170, 302, and 379. Note: The amino acid at position 302 is not identified for the Nine-banded armadillo (*Dasypus novemcinctus*), as the reference sequence contains a stretch of unknown bases between the annotated exons 3 and 4, which includes position 302. (B) Homology models of human (top panel) and cat (bottom panel) Tas1r1 VFT. The human model shows l-Glutamic acid (orange) and IMP (green) binding, while the cat model shows l-Alanine (pink) and IMP (green) binding. The residues that are different for human and cat are in cyan, and the residues important for IMP binding are in yellow. The secondary structures are in gray. The key amino acids at positions 170, 302, and 379 are highlighted in red.

Comparing the human (see Supplementary Fig. 5 for full sequence) and cat Tas1r1 VFT amino acid binding sites by sequence alignment (Fig. 2A), we confirmed the presence of 3 key amino acid substitutions for cat at positions that have been reported previously to affect ligand binding (Toda et al. 2013): ALA170GLU, ALA302ASP (McGrane et al. 2013; Toda et al. 2021), and LYS379GLY (Toda et al. 2021). Next, we generated a homology model of the cat Tas1r1 VFT domain and used this to screen for amino acid activity (Fig. 2B). Notably, the two positions at 170 and 302, which are both l-Alanine in the VFT of the human umami receptor that are replaced in cat by the acidic l-Glutamic acid and l-Aspartic acid residues, respectively, result in considerable change to the Tas1r1 VFT active site polarity and shape. Consequently, the acidic amino acids that activate the human umami receptor are predicted to be not active in cat. The difference between human and cat Tas1r1 VFT in position 379, which is LYS in human TAS1R1 and GLY in cat, is predicted to still result in IMP binding in cat.

### Nucleotides are agonists, while some l-amino acids act as enhancers, of the cat umami receptor

**Fig. 3.**
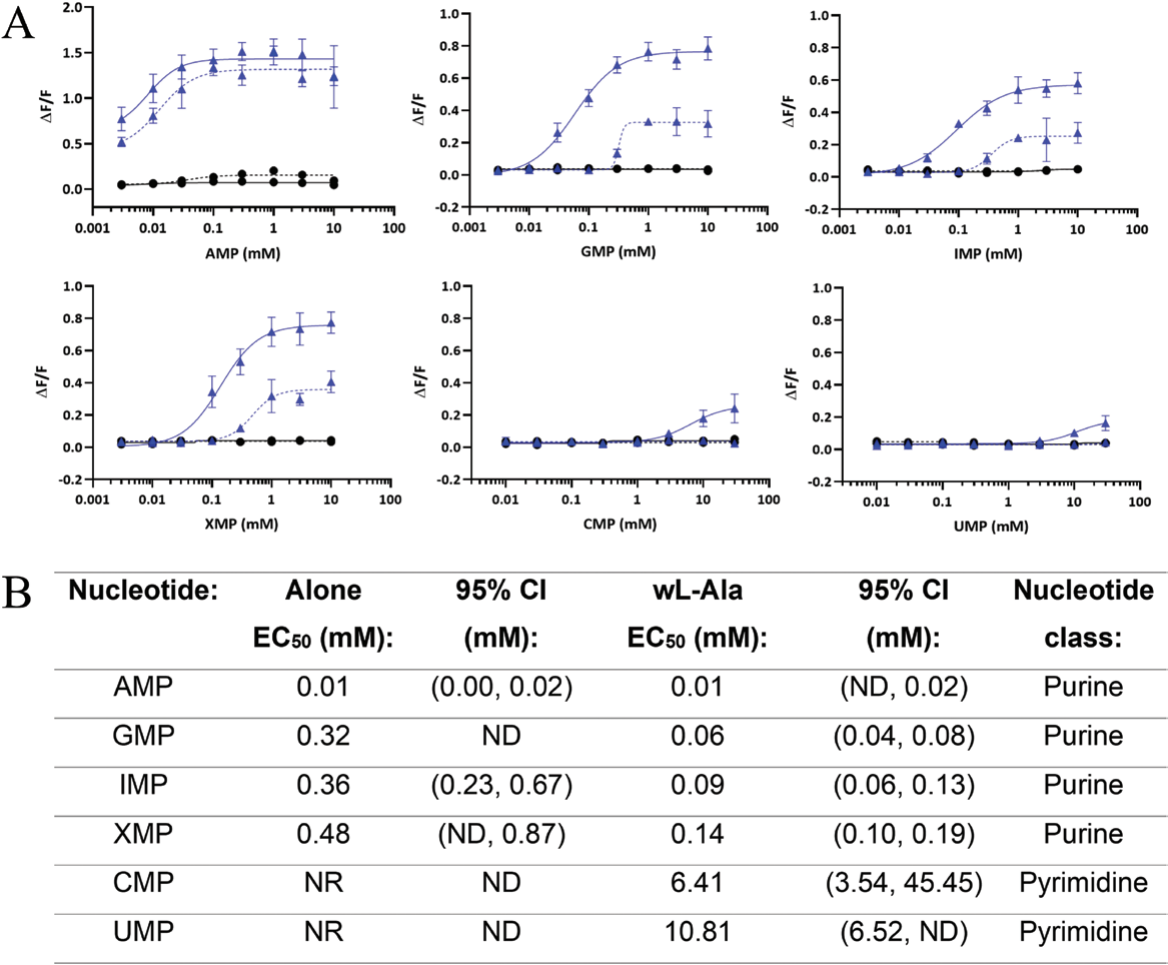
(A) In vitro concentration–response curves for 6 nucleotides arranged in ascending order of EC50 values. Change in fluorescence (ΔF/ F) is shown on the y-axis and nucleotide concentration (mM) is shown on the x-axis. AMP is Adenosine 5ʹ-monophosphate, CMP is Cytidine 5ʹ-monophosphate, GMP is Guanosine 5ʹ-monophosphate, IMP is Inosine 5ʹ-monophosphate, and UMP is Uridine 5ʹ-monophosphate. Blue broken line is cat Tas1r1-Tas1r3 without l-Alanine, blue continuous line is cat Tas1r1-Tas1r3 with 20 mM l-Alanine, black broken line is mock without l-Alanine, and black continuous line is mock with 20 mM l-Alanine. (B) Nucleotide EC50 values arranged in ascending order, with 95% confidence intervals (CI), and nucleotide chemical class. NR is no response and ND is not determined.

The purine nucleotides Adenosine 5ʹ-monophosphate (AMP), GMP, IMP, and Xanthosine 5ʹ-monophosphate (XMP) were agonists of the cat umami receptor, while there was no response obtained to the pyrimidine nucleotides Cytidine 5ʹ-monophosphate (CMP) and Uridine 5ʹ-monophosphate (UMP) when screened alone (see Fig. 3). All nucleotides were also screened in the presence of 20 mM l-Alanine to identify if there was a synergistic interaction. In this case, there was a response obtained for all the nucleotides in combination with l-Alanine, with the purine nucleotides having lower EC_50_ values compared to the pyrimidine nucleotides. Overall, the EC_50_ values obtained for the purine nucleotides in combination with l-Alanine were lower than when tested alone, indicative of the umami synergy response. Interestingly, the concentration–response curves for AMP were different to the other purine nucleotides, whereby they had lower EC_50_ values and reached a plateau earlier (both around 0.1 mM). Although the response was higher for AMP with 20 mM l-Alanine compared to AMP alone, the difference was small. There was also some response for the mock cell line without l-Alanine, which is the only instance in which this was observed.

**Fig. 4.**
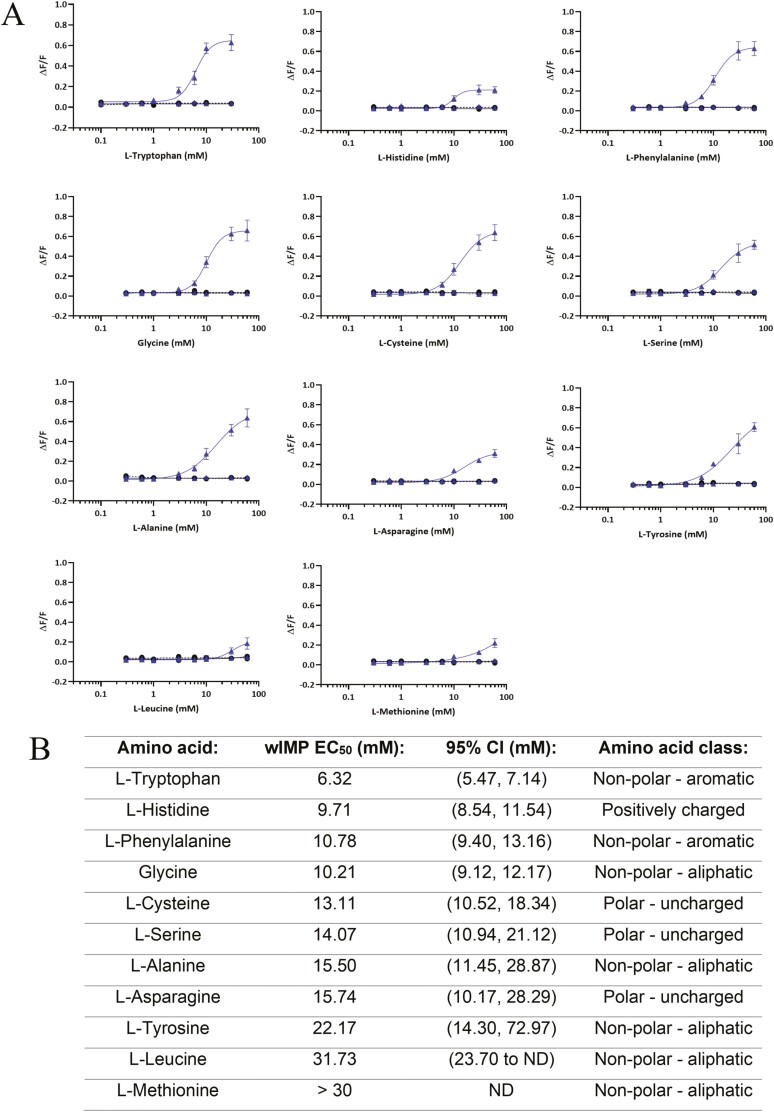
(A) In vitro concentration–response curves for 11 l-amino acids arranged in ascending order of EC50 values. Change in fluorescence (ΔF/ F) is shown on the y-axis and amino acid concentration (mM) is shown on the x-axis. Blue broken line is cat Tas1r1-Tas1r3 without IMP, blue continuous line is cat Tas1r1-Tas1r3 with 0.2 mM IMP, black broken line is mock without IMP, and black continuous line is mock with 0.2 mM IMP. B). l-amino acid EC50 values arranged in ascending order, with 95% confidence intervals (CI), and amino acid chemical class. An EC50 value for l-Methionine could not be determined due to not reaching a plateau, and value of > 30 mM was assigned. ND is not determined.

None of the 22 l-amino acids tested were agonists of the cat umami receptor (Fig. 4 and see Supplementary Fig. 6). All amino acids were also screened in the presence of 0.2 mM IMP to identify if there was a synergistic interaction. In this case, there was a response obtained for 11 of the amino acids in combination with IMP, including l-Alanine, l-Asparagine, l-Cysteine, Glycine, l-Histidine, l-Leucine, l-Methionine, l-Phenylalanine, l-Serine, l-Tryptophan, and l-Tyrosine (Fig. 4). Hence, these amino acids appear to be acting as enhancers of the umami receptor. The EC_50_ values obtained for the l-amino acids in combination with IMP were all in the mM concentration range. Only an approximated EC_50_ value is stated for l-Methionine with IMP, as the response had not reached a plateau at the highest concentration tested of 60 mM. l-Tryptophan was insoluble at 60 mM, so the maximum concentration tested in vitro was 30 mM. l-Tyrosine showed some precipitation at 30 mM and 60 mM, thus the indicated concentrations for l-Tyrosine should be considered only as approximate concentrations. As predicted, the cat umami receptor did not respond to l-Glutamic acid or l-Aspartic acid either as agonists or enhancers, which are the main amino acid agonists for the human umami receptor (see Supplementary Fig. 6 for in vitro results of all non-active amino acids). The corresponding d-amino acids were also tested using the in vitro assay, and all were found to be inactive (results are not shown). Taurine, which is a naturally-occurring sulfonic acid that is essential for cats as they lack the enzyme (sulfinoalanine decarboxylase) required to produce taurine and must therefore acquire it from their diet (Knopf et al. 1978; NRC 2006), was also screened and found to be inactive (see also Supplementary Fig. 6 for result).

### Nucleotides, amino acids, and their mixtures are appetitive for cats

**Fig. 5.**
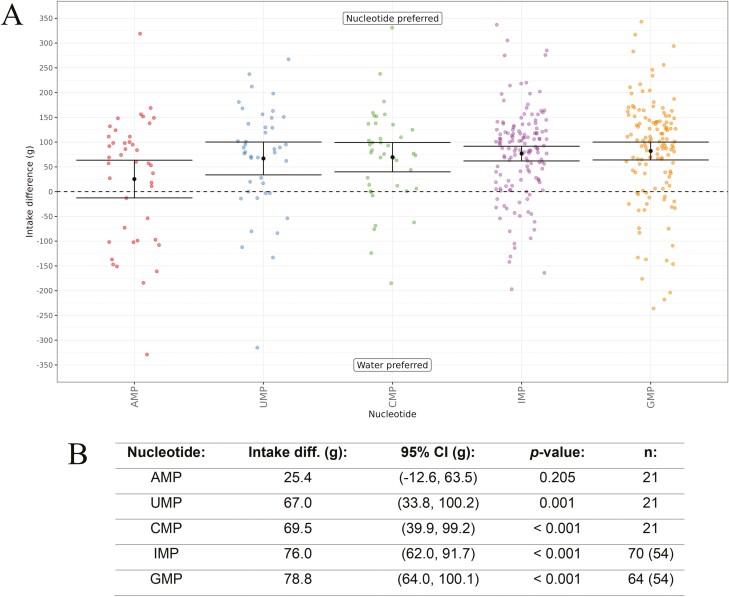
(A) In vivo response of cats to 5 nucleotides using a Water Panel. The difference in intake (g) is shown on the y-axis and the nucleotides tested are shown on the x-axis. Means are shown with 95% confidence intervals. The nucleotides are ordered from left to right, from lowest to highest difference in intake (g). All nucleotides were tested at 5 mM. (B) Nucleotide intake difference values (g) ordered from lowest to highest, with 95% confidence intervals (CI), *P*-values, and *n* values (where multiple tests were run, the number of unique cats is shown in brackets).

As nucleotides were broadly active in the in vitro cat Tas1r1-Tas1r3 assay, we first determined the behavioral response of cats to 5 of the nucleotides using a 2-bottle choice test with a panel trained to discriminate between taste compounds in water (Laffitte et al. 2021). Unfortunately, we could not derive a presumed safe intake for XMP for cats, so it was not tested here. All nucleotides were tested at a concentration of 5 mM versus water (Fig. 5). The cats had a significant preference for the nucleotides in all comparisons, except for AMP, which had no significant preference versus water.

**Fig. 6.**
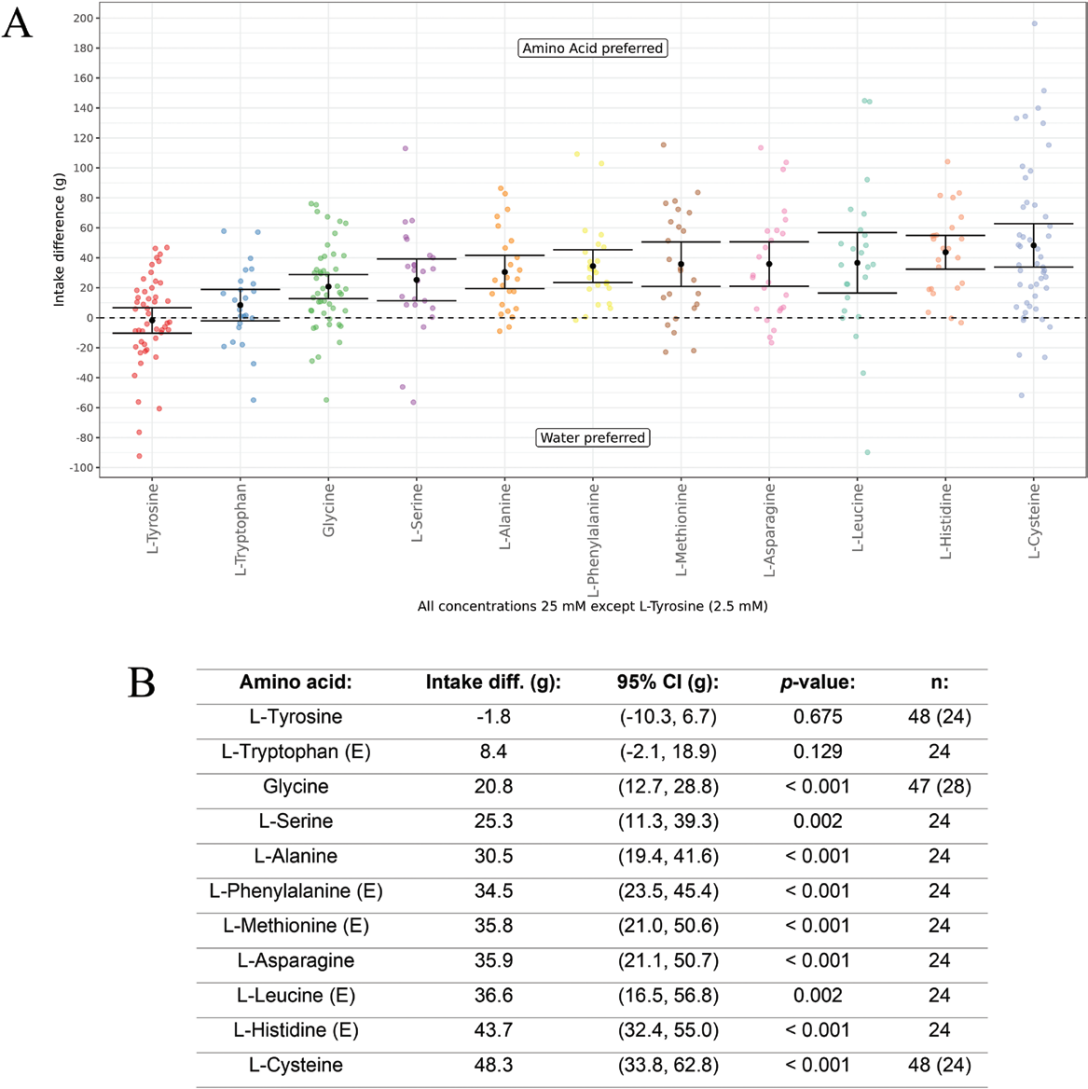
(A) In vivo response of cats to 11 l-amino acids versus water using a Water Panel. The difference in intake (g) is shown on the y-axis and the amino acids tested are shown on the x-axis. Means are shown with 95% confidence intervals. The amino acids are ordered from left to right, from lowest to highest difference in intake (g). All amino acids were tested at 25 mM, except for l-Tyrosine which was tested at 2.5 mM due to having low solubility in water. (B). Amino acid intake difference values (g) ordered from lowest to highest, with 95% confidence intervals (CI), essential amino acids for cats indicated by (E), *P*-values, and *n* values (where multiple tests were run, the number of unique cats is shown in brackets).

Having identified 11 amino acids that were active as enhancers in combination with IMP using the in vitro cat Tas1r1-Tas1r3 assay, we next tested the behavioral response of cats to these amino acids. All amino acids were tested at a concentration of 25 mM versus water, except for l-Tyrosine which we decided to test at 2.5 mM (one-tenth the concentration of the other amino acids) due to its low solubility in water (Fig. 6). Indeed, l-Tyrosine has the lowest solubility in water of all the amino acids tested (Wu 2013). The cats had a significant (*P* < 0.01) preference for the amino acid in all comparisons, except for l-Tyrosine and l-Tryptophan, for which the cats had no significant preference at the concentrations tested versus water.

**Fig. 7.**
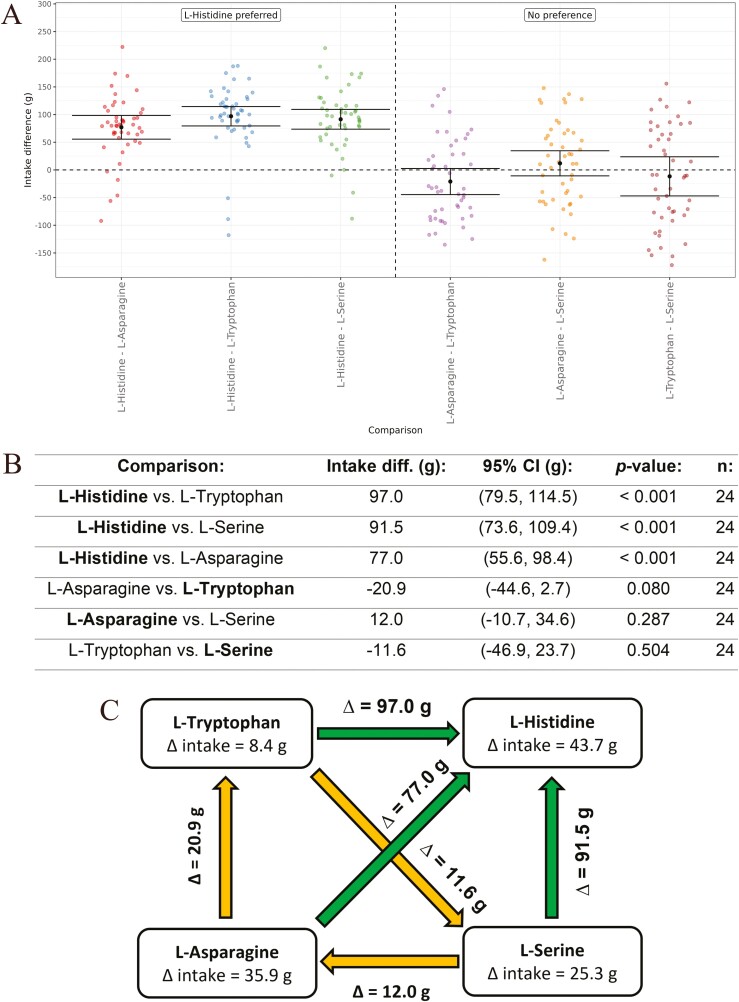
(A) In vivo response of cats to l-amino acids compared directly using a Water Panel. The difference in intake (g) is shown on the y-axis and the amino acids being compared are shown on the x-axis. Means are shown with 95% confidence intervals. The amino acids were selected to represent the range of responses obtained in vitro and in vivo. All amino acids were tested at 25 mM. (B) Amino acid intake difference values (g) ordered from lowest to highest, with 95% confidence intervals (CI), *P*-values, and *n* values. The direction of the cats’ preference for the amino acid comparison is highlighted in bold text. (C) Summary chart of the direct comparisons. The difference in intake versus water (obtained from Fig. 6) are shown in the boxes for each amino acid. A green arrow represents a significant preference for the amino acid. An orange arrow represents a non-significant preference (trend) for the amino acid. The difference in intake (g) for each comparison is shown above the arrows.

We then compared directly 4 of the amino acids to further understand their relative preference by the cats. A round-robin design was used, where the 4 amino acids were tested versus each other, leading to 6 comparisons in total. The amino acids were selected to represent the range of responses obtained in the experiments versus water (Fig. 6) and all amino acids were tested at a concentration of 25 mM (Fig. 7). Interestingly, there was a highly-significant (*P* < 0.001) preference for l-Histidine in all comparisons, while there were no significant differences obtained for any of the other comparisons. There was a directional preference for l-Tryptophan when compared with l-Asparagine that was significant at a 10% significance level (+20.9 g, *P* = 0.080).

**Fig. 8.**
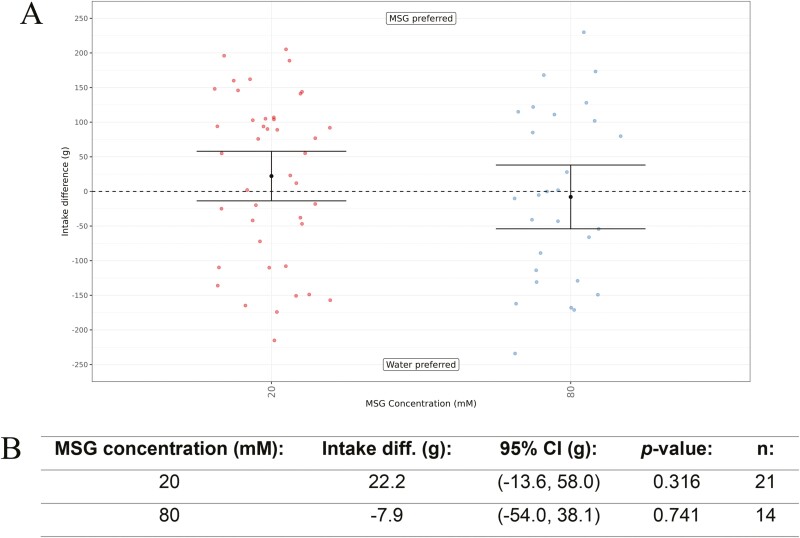
(A) In vivo response of cats to two concentrations of MSG (20 mM and 80 mM) versus water using a Water Panel. The difference in intake (g) is shown on the y-axis and the MSG concentrations tested are shown on the x-axis. Means are shown with 95% confidence intervals. (B). MSG intake difference values (g), with 95% confidence intervals (CI), *P*-values, and *n* values.

As the acidic amino acids were confirmed not to be active in the cat umami receptor assay, we also wanted to determine the behavioral response of the cats to these amino acids, as these are the main umami agonists amino acids for humans. However, it has been reported previously that cats avoided l-Glutamic acid solutions (Beauchamp et al. 1977; McGrane et al. 2013) and also l-Aspartic acid solutions due to being acidic (McGrane et al. 2013), so we instead elected to use MSG to study the response here. Hence, we tested MSG at two concentrations of 20 mM and 80 mM versus water (Fig. 8). In both cases, the cats had no significant preference for the MSG.

**Fig. 9.**
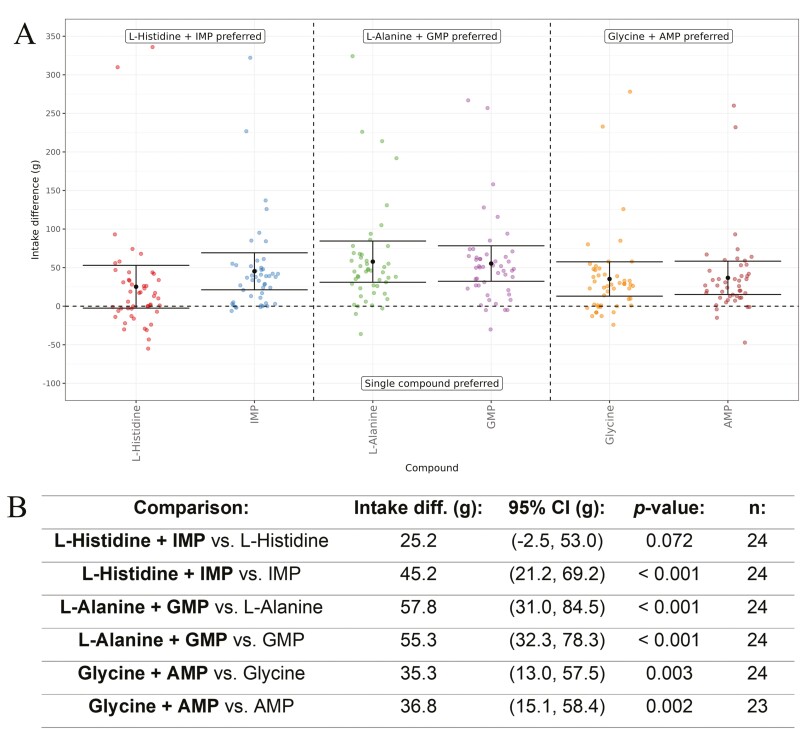
(A) In vivo response of cats to a nucleotide or l-amino acid compared directly to the same mixture using a Water Panel. The difference in intake (g) is shown on the y-axis and the nucleotide or amino acid being compared to the corresponding mixture is shown on the x-axis. Means are shown with 95% confidence intervals. All nucleotides were tested at 2.5 mM and all amino acids were tested at 25 mM. (B) Nucleotide or amino acid versus the corresponding mixture intake difference (g), with 95% confidence intervals (CI), *P*-values, and *n* values. The direction of the cats’ preference for the comparison is highlighted in bold text.

Finally, we directly compared a range of mixtures of amino acids and nucleotides to either the amino acid or the nucleotide alone. All amino acids were tested at a concentration of 25 mM and all nucleotides were tested at a concentration of 2.5 mM alone and in the mixtures (Fig. 9). As expected, there was a significant (*P* < 0.01) preference for the mixtures in all cases, except for the comparison of l-Histidine versus l-Histidine + IMP, where there was a directional preference for the mixture that was significant at a 10% significance level (+25.2 g, *P* = 0.072).

## Discussion

Striking differences in taste perception linked to dietary specialization in a range of species have now been reported, including cats (Li et al. 2005), hummingbirds (Baldwin et al. 2014), giant panda, and sea lion (Beauchamp and Jiang 2015). Indeed, the sense of taste of the domestic cat (*Felis catus*), which is an obligate carnivore, appears to have evolved to primarily detect compounds found in meat. As an obligate carnivore, we propose that umami (mediated by the Tas1r1-Tas1r3 heterodimer) is the main appetitive taste modality for the domestic cat. Here, we report the results of comprehensive studies using a range of complimentary approaches, including phylogenetics, molecular modeling, in vitro assays, and in vivo behavior, to elucidate the umami taste perception and preferences of the cat.

We constructed a phylogenetic tree based on sequence alignment of Tas1r1 for 47 mammalian species that were extracted from Ensembl (www.ensembl.org) (Fig. 2). The species were classified as carnivores, omnivores, or herbivores and 3 key binding residues linked to Tas1r1 activity of umami compounds are also shown. Previous research (Toda et al. 2013, 2021) identified key binding amino acid residues in Tas1r1 at positions 170, 302, and 379, and showed that the combination of two distinct determinants, amino acid selectivity at the orthosteric site and receptor activity modulation at the non-orthosteric sites, mediates the ligand specificity of the umami receptor (Tas1r1-Tas1r3).

The most common variation in the 3 key binding sites amongst the mammalian species included in the phylogenetic tree is EDG. This accounts for 21 of the 47 species and includes the domestic cat (*Felis catus*) and also the domestic dog (*Canis familiaris*). Of these species, 8 are carnivores, 11 are omnivores and only 2 are herbivores, so the EDG variation is more common in mammals that eat meat. Two more species (*Sus scrofa* and *Orcinus orca*) were DDG. D is an acidic amino acid, like E, and would infer similar binding properties. However, it has been reported that the 3 residues are DDG in pig and that the pig Tas1r1-Tas1r3 in vitro assay was activated by l-Glutamic acid (Toda et al. 2021) and site-directed mutagenesis would be required to determine the role the amino acid residues play in amino acid binding for these species. The EDG and DDG variations include all the carnivorous species in the phylogenetic tree, except for the Tasmanian devil (*Sarcophilus harrisii*), which is GAS. These amino acids have different chemical properties compared to EDG and would presumably result in a different binding profile, even though Tasmanian devils eat meat, but are scavengers and prefer to eat carrion (Andersen et al. 2020).

Due to the changes in two of the key amino acid binding residues in the human and cat Tas1r1 (ALA170GLU and ALA302ASP), our model (Fig. 2B) showed that the binding profile of the cat umami receptor is significantly different from the human umami receptor given the very different chemical characteristics of these amino acid substitutions. The negatively charged amino acids l-Glutamic acid and l-Aspartic acid that are ligands of the human TAS1R1 are not ligands of the cat Tas1r1 because of the prevalence of negative charges in the hinge region due to the presence of the negatively charged GLU170 and ASP302 in the cat Tas1r1, as opposed to the aliphatic ALA at positions 170 and 302 in humans. This is also supported by the analysis of single-point mutants at these positions in mouse Tas1r1 and human TAS1R1 (Toda et al. 2013). For the amino acids that are umami-active for cats (such as l-Alanine), the amino acid groups are positioned deep in the active site of the Tas1r1 VFT with many charged, polar, and hydrophobic interactions. The positioning of IMP into human and cat Tas1r1 VFT followed the modeling (Zhang et al. 2008) for human TAS1R1 (Fig. 2B), where it was experimentally proven that the amino acids HIS71, ARG277, SER306, and HIS308 are critical for IMP binding in human TAS1R1. It was also conjectured (Zhang et al. 2008) that those residues were co-ordinating the phosphate group of IMP. There is a difference between human and cat Tas1r1 VFT in position 379, which is LYS in human TAS1R1 and GLY in cat.

When screened alone, the purine nucleotides (AMP, GMP, IMP, and XMP) were agonists of the cat umami receptor, while there was no response obtained for the pyrimidine nucleotides (CMP and UMP) (Fig. 3). These results are consistent with those recently reported for IMP and GMP, which were identified as agonists of the cat umami receptor (Toda et al. 2021). There appears to be a difference in the sensitivity of the cat Tas1r1-Tas1r3 in vitro assays used, whereby Toda et al.’s assay started to respond to IMP and GMP at around 0.01 mM compared to ours at around 0.1 mM. This may be due to differences in the G-protein chimeras used in the assays: rat or mouse Gα15 (see Supplementary Fig. 1). There was a low level of activity in the mock cells as the concentration of AMP increased, but this was much lower than the activity of cells with cat Tas1r1-Tas1r3. AMP could not be examined in Toda et al.’s assay due to unexpected non-specificity of cellular responses. When screened in the presence of 20 mM l-Alanine, the response of the purine nucleotides was enhanced, with increased signal amplitude and lower EC_50_ values, except for AMP which had only a slightly higher response with l-Alanine compared to without. There was also a small response obtained for the pyrimidine nucleotides with l-Alanine. These results are consistent with previous research on the synergism of nucleotides and l-amino acids using rat taste nerve responses, where 5ʹ-nucleotides with a pyrimidine base such as UMP and CMP had only a small or no enhancing effect with amino acids (Yoshii et al. 1986).

Although the cat umami receptor did not respond to l-amino acids alone, 11 of those tested enhanced the response to IMP (Fig. 4). Amino acids can be classified into classes based on the chemical characteristics of their R groups (Wu 2013; Berg et al. 2015). The amino acids that were umami-enhancers for cats covered all these classes, except for negatively-charged amino acids, which were expected to be inactive due to the substitutions at positions 170 and 302 in the cat Tas1r1 described above.

The in vitro binding of the mouse (*Mus musculus*) umami receptor (Tas1r1-Tas1r3) has been reported for 19 proteinogenic l-amino acids (all except l-Tyrosine due to being insoluble at the concentration of 50 mM tested) and a selection of d-amino acids (Nelson et al. 2002). Even though the umami receptor of the domestic cat and the mouse both have the same variation in the 3 key binding sites (i.e. EDG), the binding profiles are not the same. The mouse umami receptor responded to a range of l-amino acids as agonists. In the presence of a nucleotide (2.5 mM IMP), the response was further enhanced, and indeed there was now a response obtained for nearly every l-amino acid in combination with IMP. On the other hand, the cat umami receptor did not respond to any l-amino acids as agonists. Instead, it responded to the purine nucleotides as agonists and the response was enhanced in the presence of some l-amino acids. The concentration of IMP we used of 0.2 mM was much lower than that used with the mouse work of 2.5 mM. The concentration of the amino acids tested with the mouse Tas1r1-Tas1r3 was 50 mM, similar to the maximum l-amino acid concentration of up to 60 mM we used for the concentration–responses with the cat umami receptor. The difference in the binding profiles of the mouse and cat Tas1r1-Tas1r3 may be related to their dietary habits, with mouse being an omnivore and cat being an obligate carnivore. Further research using site-directed mutagenesis would help to elucidate the cause of the differences in the activity of the mouse and cat umami receptors. A key difference in the response of the cat in vitro assay compared to the behavioral tests is the requirement of a nucleotide to obtain a response from the l-amino acids in the in vitro assay, whereas the cats had a significant behavioral preference for most of the l-amino acids when tested at 25 mM without a nucleotide added (Fig. 6). AMP has been identified in human saliva, albeit at a much lower concentration of 0.99 µM (Kochańska et al. 2000) compared to the concentration of 0.2 mM IMP used in our assay. Hence it is possible, although not known, that nucleotides when combined with free l-amino acids, enhance umami taste and allow the cats to detect these amino acids.

The cats had a significantly higher intake of all the nucleotide solutions compared to water (Fig. 5), except for AMP which had no significant difference in intake (g) (*P* = 0.205). Interestingly, there were some differences in the activity of the nucleotides obtained from the in vitro assay and the behavioral tests. In the in vitro assay, when screened alone, there was only a response for the purine nucleotides and no response for the pyrimidine nucleotides (Fig. 3). However, in the behavioral results, the intake difference for IMP and GMP (purine nucleotides) were similar to those obtained for UMP and CMP (pyrimidine nucleotides) (Fig. 5). The behavior tests used an 18-h exposure period, which may not differentiate taste versus post-ingestive cues for the responses. To further confirm our results, we conducted shorter 1-h exposure period behavioral tests with a subset of the nucleotides (see Supplementary Fig. 7). In the 18-h exposure test, there was no significant difference in intake obtained for AMP (*P* = 0.205); however, it was significant in the 1-h exposure test (*P* < 0.001). IMP had a significant difference in intake in both the 18-h and 1-h exposure period tests (both *P* < 0.001).

The cats had a significantly higher intake of all the amino acid solutions compared to water (Fig. 6), except for l-Tyrosine and l-Tryptophan, where there was no significant difference in intake (g) (*P* = 0.675 and *P* = 0.129, respectively). The result obtained for l-Tyrosine is likely due to the lower concentration tested (2.5 mM) due to its low solubility. Although the difference in intake for l-Tryptophan was not statistically significant, there was a directional preference in favor of the amino acid. Indeed, we also conducted shorter 1-h exposure period behavioral tests with a subset of the l-amino acids (see Supplementary Fig. 8). In the 1-h exposure tests, all the amino acids tested had a significant difference in intake (*P* < 0.001 for all tests), including l-Tryptophan. l-Tyrosine was not repeated using the 1-h exposure. Overall, the results obtained with both the 18-h and 1-h exposure period tests were very similar in terms of the direction and ordering of the preferences, as well as significance.

The behavioral response of domestic cats for some amino acids has been reported using choice tests previously. White and colleagues used a 24-h repeat exposure to study the response of cats to solutions of several amino acids in 50 mM saline (NaCl) versus 50 mM saline (White and Boudreau 1975). Two of the amino acids, l-Histidine and l-Tryptophan (both tested at 0.5, 5.0, and 50.0 mM), were also tested in our work. l-Histidine, which was selected as it caused an increase in spike output form the geniculate ganglion chemoresponsive group II units was preferred, in agreement with our results. l-Tryptophan, which was selected as it decreased group II discharge and was avoided by the cats in saline solution. However, it was also noted that cats showed a reduction in group II discharge in distilled water with most responses being < 10% of maximum discharge. Beauchamp and colleagues used a 1-h exposure time with repeat exposure to study the response of cats to solutions of Glycine and l-Alanine (both tested at 10, 20, 40, and 80 mM) versus water (Beauchamp et al. 1977). Surprisingly, the cats were indifferent to all concentrations of Glycine studied, which is opposite to our results where 25 mM was significantly preferred versus water. The tests with l-Alanine were repeated twice. There was a preference for l-Alanine in the first test and then no preference was obtained in the second test. A reason for the difference in responses was not proposed, and it was concluded that l-Alanine may be preferred to water. We obtained a significant preference for 25 mM l-Alanine versus water in our experiment, which is in agreement with the first result obtained. Additionally, it has been reported that cats avoided all concentrations of l-Glutamic acid studied (10, 20, 40, and 80 mM) (Beauchamp et al. 1977), which is why we decided to study the cat’s response to MSG (Fig. 8), rather than l-Glutamic acid. The reason for the avoidance of l-Glutamic acid (and also l-Aspartic acid) is due to the acidic solutions obtained. Both amino acids were not active in the cat in vitro assay (either with or without IMP) and the cats did not have a significant preference for MSG over a similar concentration range used by Beauchamp and colleagues.

Tuna is a commonly used raw material and flavor in processed cat foods and is renowned for being highly palatable; however, the reason(s) for it being so palatable has not yet been identified. We propose that this is due to the specific combination of the high IMP and free l-Histidine contents of tuna, which produces a strong umami taste synergy that is highly preferred by cats. The purine nucleotide IMP is a strong agonist of the cat umami receptor (Toda et al. 2021) (Fig. 3) and highly preferred by cats in our behavioral assay (76.0 g, Fig. 5). Similarly, the amino acid l-Histidine acts as an enhancer of the cat umami receptor in combination with IMP (Fig. 4) and is one of the most-preferred amino acids for cats. It had the second-highest intake difference versus water (43.7 g, Fig. 6) and was also significantly preferred (*P* < 0.001) in all 3 direct comparisons of amino acids (Fig. 7). The IMP content of Yellowfin and Albacore tuna has been reported to be 8.4 mM—around 10 mM (Suyama et al. 1986; Price et al. 1991; Zhang et al. 2019) and the free l-Histidine content of Yellowfin and Skipjack tuna has been reported to be 7.9–89.5 mM (Abe 1983; Suyama et al. 1986; Zhang et al. 2019). Notably, IMP is the highest concentration nucleotide and l-Histidine is the highest concentration free amino acid present in tuna (Abe 1983; Suyama et al. 1986; Price et al. 1991; Zhang et al. 2019), with white meat having a higher free l-Histidine content than red meat (Abe 1983; Suyama et al. 1986; Antoine et al. 2001). Tuna white meat is primarily utilized in human foods, while tuna red meat is currently used in fertilizers and animal feeds (Parvathy et al. 2018). In comparison, American red snapper, as an example of a non-tuna fish (Antoine et al. 2001), and rat muscle meat, as an example of a typical prey food for cats (Abe 1983), both have a much lower l-Histidine content, while other muscle meat sources, including pork, lamb, turkey, beef, and chicken had no free l-Histidine detected (Triki et al. 2018). A summary of the IMP and free l-Histidine concentrations in these meat sources is presented in Table 1, with concentrations in the original units from each paper and also converted in mM concentration for ease of comparison with the units used here. The concentrations of IMP and free l-Histidine detected in a range of tuna species are well above the EC_50_ value for l-Histidine in combination with 0.2 mM IMP (9.71 mM, Fig. 4) for white meat and similar to the EC_50_ value for red meat. In addition, since cats had a significant behavioral preference for IMP at 5 mM (Fig. 5) and l-Histidine at both 25 mM (Fig. 6) and 100 mM (McGrane et al. 2013), the concentrations of IMP and free l-Histidine in tuna would also be preferred. Hence, the specific combination of the high IMP and free l-Histidine contents of tuna could indeed be a key driver of taste preference for cats of this highly palatable raw material.

**Table 1.** Concentrations of IMP free l-Histidine determined for various meat sources collated from the literature.

Meat source	IMP concentration
**Yellowfin tuna (** **Suyama et al. 1986**)	292 mg/ 100g **(8.4 mM)**
**Albacore tuna (** **Price et al. 1991**)	Around 10 µmol/ g **(10 mM)**
**Yellowfin tuna (** **Zhang et al. 2019**)	343 mg/ 100 g **(9.9 mM)**

Meat source	Free l-Histidine concentration
**Skipjack tuna** **Skipjack tuna** (**Abe 1983**)	White meat: 89.5 µmol/ g wet weight **(89.5 mM)** Red meat: 17.3 µmol/ g wet weight **(17.3 mM)**
**Yellowfin tuna** **Yellowfin tuna** **Rat muscle** (**Suyama et al. 1986**)	White meat: 947 mg/ 100 g **(61.0 mM)** Red meat: 123 mg/ 100 g **(7.9 mM)** 0.310 µmol/ g wet weight **(0.310 mM)**
**Mahi-mahi tuna** **Mahi-mahi tuna** **Yellowfin tuna** **Yellowfin tuna** **American red snapper** (**Antoine et al. 2001**)	^a^White meat: 308.8 mg/ 100 g **(19.9 mM)** ^a^Red meat: 223.4 mg/ 100 g **(14.4 mM)** ^a^White meat: 956.6 mg/ 100 g **(61.7 mM)** ^a^Red meat: 378.7 mg/ 100 g **(24.4 mM)** ^b^3.33 mg/ 100 g **(0.21 mM)**
**Yellowfin tuna** (**Zhang et al. 2019**)	437 mg/ 100 g **(28.2 mM)**
**Pork/ Lamb/ Beef/ Turkey leg muscle, Chicken breast** (**Triki et al. 2018**)	**Not detected**

Values are quoted in the original units from each paper and have also been converted in mM concentration for ease of comparison with the units used in this paper. Concentrations in mM were calculated assuming 1 kg = 1 L, using a molecular weight for IMP of 348.21 g/ mol and a molecular weight for l-Histidine of 155.15 g/ mol.

Concentrations of IMP and L-Histidine in the original units from each paper have also converted into mM concentration for ease of comparison with the units used here and are shown in bold.

^a^Calculated average of 4 samples.

^b^Calculated average of 6 samples.

Ten of the 20 proteinogenic amino acids plus Taurine (which is often included with the amino acids, but has a sulfonic acid group rather than the carboxyl group) are essential for the domestic cat (Knopf et al. 1978; NRC 2006). Five of the 11 essential amino acids (l-Histidine, l-Leucine, l-Methionine, l-Phenylalanine, and l-Tryptophan) are also enhancers of the umami receptor for cats. Although l-Methionine and l-Phenylalanine are essential within the diet of cats, l-Cysteine (which can be synthesized from l-Methionine) and l-Tyrosine (which can be synthesized from l-Phenylalanine) are also grouped with the essential amino acids as, if provided in sufficient quantities, they help free-up l-Methionine and l-Phenylalanine for other functions. Interestingly, both l-Methionine and l-Phenylalanine are also umami enhancers for cats. The essential amino acids being umami enhancers makes sense, as these amino acids are generally appetitive for cats and their presence in meat sources may promote intake and be a signal for protein quality. In the behavioral test where umami-active amino acids with a range of EC_50_ values were compared directly in a round robin design (Fig. 7), l-Histidine is significantly preferred to the other amino acids in all comparisons. It is possible, although not known specifically for cats, that the other amino acids that were compared to l-Histidine may also have other taste qualities such as bitter, which can affect their palatability. Even so, this result further demonstrates that l-Histidine is one of the most highly palatable amino acids for cats, which could be due to a combination of both taste (it is an amino acid umami enhancer for cats) and nutrition (it is an essential amino acid for cats).

In the final test, we examined the cat’s behavioral response for mixtures of l-amino acids and nucleotides by directly comparing a range of mixtures to either the corresponding l-amino acid or the nucleotide alone (Fig. 9). The cats had a significant preference for the mixtures in all cases, except for the comparison of l-Histidine versus l-Histidine + IMP, where there was a directional preference for the mixture (*P* = 0.072). The umami synergy between amino acids and nucleotides is well known for humans (Yamaguchi and Ninomiya 2000) and also appears to be active for cats. Looking at the results from the behavioral tests in detail, it also is interesting to note that some cats have a very high preference for all the mixtures (in some cases they drink nearly all 350 g of the solution offered), but there are no cats that have such high intakes for the amino acid or nucleotide alone (the maximum difference in intake was around 50 g for l-Histidine). The directional preference obtained for the mixture of l-Histidine and IMP may be due to l-Histidine being both an enhancer of the cat umami receptor as well as an essential amino acid for cats, as discussed previously.

These results provide an insight into the fascinating sensory world of the domestic cat. Nucleotides were found to be agonists of the cat umami receptor, and while no l-amino acids were agonists, some were found to enhance the umami response in combination with a nucleotide. Indeed, nucleotides, free l-amino acids, and their mixtures were highly preferred by cats. Having an umami receptor that is adapted to detect a broad range of nucleotides and amino acids may help to promote protein intake and be a signal for protein quality for cats. As an obligate carnivore, we propose that the umami receptor (Tas1r1-Tas1r3) is the main appetitive taste modality for the domestic cat, enabling them to detect key flavor compounds in meat.

## Supplementary Material

bjad026_suppl_Supplementary_Data

## Data Availability

The data underlying this article will be shared on reasonable request to the corresponding author.
