# Supplementary material for: Umami taste perception and preferences of the domestic cat (Felis catus), an obligate carnivore
Source: Chem Senses. 2023 Aug 8;48:bjad026. doi: 10.1093/chemse/bjad026 (PMC10468298; doi:10.1093/chemse/bjad026)
Supplement: bjad026_suppl_Supplementary_Data [file bjad026_suppl_supplementary_data.docx]

**Umami taste perception and preferences of the domestic cat (*Felis catus*), an obligate carnivore – Supplementary Data**

Scott J. McGrane^1^ (ORCiD: 0000-0002-9276-8644), Matthew Gibbs^1^ (ORCiD: 0000-0002-5366-0809), Carlos Hernangomez de Alvaro^1^ (ORCiD: 0000-0002-5050-0148), Nicola Dunlop^1^ (ORCiD: Not available), Marcel Winnig^2^ (ORCiD: 0000-0002-5306-5778), Boris Klebansky^3^ (ORCiD: 0009-0009-7519-208X), Daniel Waller^1^ (ORCiD: Not available).

^1^Waltham Petcare Science Institute, Freeby Lane, Waltham-on-the-Wolds, Melton Mowbray, Leicestershire, LE14 4RT, United Kingdom.

^2^AXXAM GmbH, Byk-Gulden Str.2, 78467 Constance, Germany.

^3^BioPredict, Inc., 4 Adele Avenue, Demarest, NJ 07627, USA.

Correspondence to be sent to: [scott.mcgrane@effem.com](mailto:scott.mcgrane@effem.com)

**Comparison of G-protein chimera amino acid sequences**

mGα15i1 used in our work:

MARSLTWGCCPWCLTEEEKTAARIDQEINRILLEQKKQEREELKLLLLGPGESGKSTFIKQMRIIHGVGYSEEDRRAFRLLIYQNIFVSMQAMIDAMDRLQIPFSRPDSKQHASLVMTQDPYKVSTFEKPYAVAMQYLWRDAGIRACYERRREFHLLDSAVYYLSHLERISEDSYIPTAQDVLRSRMPTTGINEYCFSVKKTKLRIVDVGGQRSERRKWIHCFENVIALIYLASLSEYDQCLEENDQENRMEESLALFSTILELPWFKSTSVILFLNKTDILEDKIHTSHLATYFPSFQGPRRDAEAAKSFILDMYARVYASCAEPQDGGRKGSRARRFFAHFTCATDTQSVRSVFKDVRDSVLARYLD**DCGLF**

rGα15i2 used by (Toda et al., 2021):

MARSLTWGCCPWCLTEEEKTAARIDQEIN**K**ILLEQKKQER**G**ELKLLLLGPGESGKSTFIKQMRIIHG**A**GYSEEDRRAFRLL**V**YQNIFVSMQAMI**E**AMDRLQIPFSRPDSKQHASLVMTQDPYKVS**S**FEKPYAVAMQYLWRDAGIRACYERRREFHLLDSAVYYLSHLERI**A**ED**D**YIPTAQDVLRSRMPTTGINEYCFSV**Q**KTKLRIVDVGGQ**K**SER**K**KWIHCFENVIALIYLASLSEYDQCLEEN**S**QENRM**K**ESLALFSTILELPWFKSTSVILFLNKTDILEDKIHTSHLA**S**YFPSFQGPRRDAEAAK**R**FILDMYARVYASCAEP**H**DGGRKGSRARR**L**FAHFTCATDT**H**SVRSVFKDVRDSVLARYLD**DCGLF**

**Supplementary Figure 1.** Comparison of the G-protein chimera amino acid sequences for mGα15i1 used in our work and rGα15i2 used by (Toda et al., 2021). Differences in the amino acid sequences of the two G-protein chimeras are highlighted in bold red text in the sequence for rGα15i2. The last 5 amino acids in mGα15i1 and rGα15i2 are the same and are highlighted in bold blue text in both sequences. Sequence length is 374, sequence identity is 356/ 374 (95.2%), and sequence similarity is 366/ 374 (97.9%).

***Tas1r1* expression in cat fungiform papillae RT-PCR gels**

**
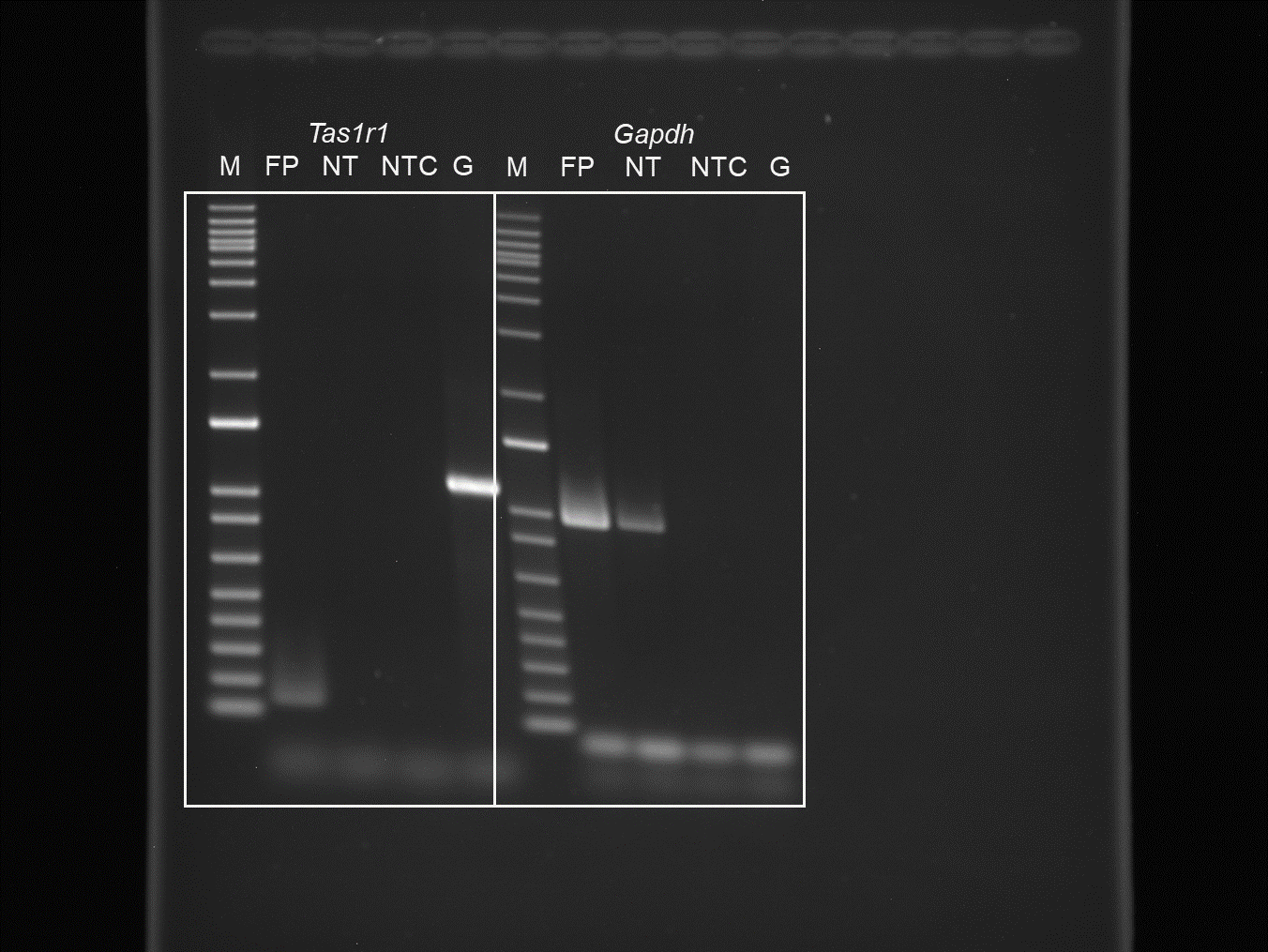
**

**Supplementary Figure 2.** Full gel corresponding to Figure 1 in the manuscript, with *Tas1r1* expression (white box left hand side) and *Gapdh* expression (white box right hand side). M = molecular size marker, FP = cat fungiform papillae, NT = non-taste epithelial tissue, NTC = no-template controls, and G = genomic DNA.

**Cat breeds used to derive reference sequences for Tas1r1 and Tas1r3**

| **Cat breed:** | **Number:** |
| --- | --- |
| Domestic Shorthair | 486 |
| Abyssinian | 4 |
| American Curl | 1 |
| Bengal | 5 |
| Japanese Bobtail | 5 |
| Chartreux | 6 |
| Norwegian Forest cat | 1 |
| Chinchilla | 1 |
| Exotic Shorthair | 8 |
| Maine Coon | 13 |
| Oriental | 6 |
| Persian | 3 |
| Birman | 8 |
| Siamese | 4 |
| Singapore | 1 |
| Somali | 7 |
| Unknown | 3 |

**Supplementary Table 1.** Breakdown of 562 cat breeds used to derive reference sequences for Tas1r1 and Tas1r3 used in *in silico* models and *in vitro* assay.

**Cat *Tas1r1* nucleic acid sequence**

ATGTCACTCCCGGCGGCTCACCTGGTCGGCCTGCAGCTCTCCCTCTCCTGCTGCTGGGCTCTCAGCTGCCACAGCACAGAGACGTCTGCCGACTTCAGCCTCCCTGGGGATTACCTCCTCGCAGGTCTGTTCCCTCTGCACTCTGACTGTCCGGGCGTGAGGCACCGGCCCACGGTGACCCTCTGTGACAGGCCCGACAGCTTCAACGGTCACGGCTACCACCTCTTCCAGGCCATGCGGTTTGGCATCGAGGAGATAAACAACTCCACGGCCCTCCTGCCGAACGTCACCCTGGGATACCAGCTGTACGACGTGTGCTCGGAGTCTGCCAACGTGTATGCCACACTAAACGTGCTCTCCCTGCTGGGGACACATCACGTAGAGATCCGAGCAGACCCTTCCCACTATTCGCCCGCCGCCCTGGCTGTCATTGGGCCTGACACCACCAACCACGCAGCCACCACTGCAGCCCTGCTGAGCCCCTTCCTGGTGCCCCTGATCAGCTACGAGGCCAGCAGCGTGACGCTCGGAGTGAAGCGGCATTACCCCTCGTTTCTGCGCACCATCCCCAGCGACAAGCACCAGGTGGAGGCCATGGTGCTGCTGCTGCAGAGCTTCGGGTGGGTCTGGATCTCGGTGGTCGGCAGCGACGGCGACTACGGGCAGCTGGGGGTGCAGGCGCTGGAGGAGCAGGCCACCCAGCAGGGCATCTGCGTTGCCTTCAAGGACATCATCCCCTTCTCTGCCCGGCCGGGCGACGAGAGGATGCAGGGCATCATGCACCACCTGGCCCGAGCGAGGACCACCGTTGTGGTCGTTTTCTCCAGCAGGCAGCTGGCCAGGGTGTTCTTTGAGTCGGTGGTGCTGGCCAACCTGACTGCCAAGGTGTGGATCGCCTCAGAAGACTGGGCCATCTCTAGACACATCAGCAATGTGCCCGGGATCCAGGGCATTGGCACAGTGCTGGGTGTGGCCATCCAGCAGAGGCTTGTCCCTGGCCTGAAGGAGTTTGAAGAGGCCTATGTCCAGGCAGATAAGGGGGCCCCTGGGCCTTGCTCCAGGACCTCCGAGTGCAGCAGCAACCAGCTCTGTAGAGAGTGTCGGGCTTTCACGGCAGAGCAGATGCCCACGCTCGGGGCATTCTCCATGAGCTCTGCTTATAACGCCTACCGGGCAGTCTACGCAGTGGCCCATGGCCTCCACCAGCTCCTGGGCTGTGCCTCTGGAGCCTGTTCCAGGGACCGAGTCTACCCCTGGCAGCTTCTGGAGCAGATCCGCAAGGTGAATTTCCTCCTACACAAGGACACCGTGAGGTTTAATGACAACGGGGACCCTCTCAGTGGCTACGACATAATTGCCTGGGACTGGAGTGGCCCCAAGTGGAACTTCAGGGTCATTGGCTCCTCCATGTGGCCTCCAGTTCAGCTGGACATAAATAAAACCAAAATCCGGTGGCACGGGAAGGACAACCAGGTGCCAAAGTCTGTGTGCTCCAGCGACTGCCTCGAAGGGCACCAGCGAGTGATTTCGGGTTTCTACCACTGTTGCTTTGAGTGTGTGCCCTGTGAGGCCGGGAGCTTCCTCAACAAGAGCGACCTCCACAGCTGCCAGCCTTGTGGGAAAGAAGAGTGGGCACCCGCGGGAAGTGAAACCTGCTTTCCACGCACCGTGGTGTTTTTGACTTGGCACGAGACCATCTCTTGGGTGCTGCTGGCAGCTAATACGTTGCTGCTGCTGCTGGTGACTGGGACTGCTGGCCTGTTTGCCTGGCACTTAGACACCCCTGTGGTGAAGTCCGCTGGGGGCCGACTGTGCTTCTTCATGCTGGGCTCCCTGGCAGGGGGCAGCTGTGGGCTCTACGGCTTTTTTGGGGAGCCCACGCTGCCCACATGCTTGTTGCGCCAAAGCCTCCTTGCCCTGGGTTTTGCCATCTTCCTGTCCTGCCTGACCATCCGCTCCTTCCAACTGGTCTTCATCTTCAAGTTTTCTGCCAAGGTACCCACCTTCTACCGTGCCTGGGTCCAAAACCACGGTCCTGGCCTATTTGTGGTGATCAGCTCAATGGCCCAGCTGCTCATCTGTCTAACTTGGCTGGCGGTGTGGACCCCACTGCCCACCAGGGAGTACCAGCGCTTCCCTCAGCTGGTGGTGCTTGATTGCACAGAGGCCAACTCACCGGGCTTCATGTTGGCTTTCGCCTACAATGGCCTCCTGTCCGTCAGCGCCTTTGCCTGCAGCTACCTGGGCAAGGACCTGCCAGAGAACTACAACGAGGCCAAATGTGTCACTTTTAGTCTGCTGCTCAACTTCGTGTCCTGGATTGCCTTCTTCACCACGGCCAGCGTCTACCAGGGCAAGTACTTGCCCGCGGTCAACGTGCTGGCGGCGCTGAGCAGCCTGAGTGGCGGCTTCAGCGGTTATTTCCTCCCCAAGTGCTACGTGATCCTGTGCCGCCCAGATCTCAACAGCACAGAGCACTTCCAGGCCTCCATCCAGGAGTACACGAGGCGCTGCGGCTCCACCTGA**Cat Tas1r1 amino acid sequence**

MSLPAAHLVGLQLSLSCCWALSCHSTETSADFSLPGDYLLAGLFPLHSDCPGVRHRPTVTLCDRPDSFNGHGYHLFQAMRFGIEEINNSTALLPNVTLGYQLYDVCSESANVYATLNVLSLLGTHHVEIRADPSHYSPAALAVIGPDTTNHAATTAALLSPFLVPLISYEASSVTLGVKRHYPSFLRTIPSDKHQVEAMVLLLQSFGWVWISVVGSDGDYGQLGVQALEEQATQQGICVAFKDIIPFSARPGDERMQGIMHHLARARTTVVVVFSSRQLARVFFESVVLANLTAKVWIASEDWAISRHISNVPGIQGIGTVLGVAIQQRLVPGLKEFEEAYVQADKGAPGPCSRTSECSSNQLCRECRAFTAEQMPTLGAFSMSSAYNAYRAVYAVAHGLHQLLGCASGACSRDRVYPWQLLEQIRKVNFLLHKDTVRFNDNGDPLSGYDIIAWDWSGPKWNFRVIGSSMWPPVQLDINKTKIRWHGKDNQVPKSVCSSDCLEGHQRVISGFYHCCFECVPCEAGSFLNKSDLHSCQPCGKEEWAPAGSETCFPRTVVFLTWHETISWVLLAANTLLLLLVTGTAGLFAWHLDTPVVKSAGGRLCFFMLGSLAGGSCGLYGFFGEPTLPTCLLRQSLLALGFAIFLSCLTIRSFQLVFIFKFSAKVPTFYRAWVQNHGPGLFVVISSMAQLLICLTWLAVWTPLPTREYQRFPQLVVLDCTEANSPGFMLAFAYNGLLSVSAFACSYLGKDLPENYNEAKCVTFSLLLNFVSWIAFFTTASVYQGKYLPAVNVLAALSSLSGGFSGYFLPKCYVILCRPDLNSTEHFQASIQEYTRRCGST*

**Supplementary Figure 3.** Cat *Tas1r1* nucleic acid sequence and cat Tas1r1 amino acid sequence.

**Cat *Tas1r3* nucleic acid sequence**

ATGCCCGGCCTCGCTCTCCTGGGCCTCACGGCTCTCCTGGGCCTCACGGCTCTCTTGGACCACGGGGAGGGCGCAACGTCCTGCTTGTCACAGCAGCTCAGGATGCAGGGGGACTATGTGCTGGGTGGGCTCTTCCCTCTGGGCTCTGCCGAGGGTACAGGTCTTGGCGACGGGCTGCAGCCCAATGCCACCGTGTGCACCAGGTTCTCGTCTCTGGGCCTGCTCTGGGCGCTGGCCGTGAAGATGGCGGTGGAGGAGATCAACAACGGGTCGGCCCTGCTGCCCGGGCTGCACCTGGGCTATGACCTCTTTGACACGTGTTCAGAGCCCATGGTGGCCATGAAGCCCAGCCTCGTGTTCATGGCCAAAGCAGGCAGCTGCAGCATTGCCGCCTACTGCAATTACACACAGTACCAGCCCCGCGTGCTGGCCGTCATCGGGCCCCACTCGTCTGAGCTCGCCCTCGTCACCGGCAAGTTCTTCAGCTTCTTCCTTGTGCCTCAGGTCAGCTACGGCGCCAGCACCGACCGGCTGAGCAACCGGGAGATCTTCCCGTCCTTCTTCCGCACGGTGCCCAGCGACCAGGTGCAGGTGGCGGCCATGGTGGAGCTGCTGCAGGAGCTCGGCTGGAACTGGGTGGCGGCGGTGGGTAGTGACGACGAGTATGGCCGGCAGGGCCTGAGCCTCTTCTCCGGCCTGGCCAGCGCCAGGGGCATCTGCATCGCGCATGAGGGCCTGGTGCCACTGCCGCCAGGCAGCCTGCGGCTGGGCGCCCTACAGGGCCTGCTGCGCCAGGTGAACCAGAGCAGCGTGCAGGTGGTGGTGCTGTTCTCCTCCGCCCACGCGGCCCGCACCCTCTTCAGCTACAGCATCCGCTGCAAGCTCTCACCCAAGGTGTGGGTGGCCAGCGAGGCCTGGCTGACCTCAGACCTGGTCATGACGCTGCCCGGCATGCCTGGGGTGGGCACCGTGCTGGGCTTCCTGCAGCAGGGCGCCCCGATGCCGGAGTTCCCATCCTACGTGCGGACCCGCCTGGCCCTGGCCGCTGACCCTGCCTTCTGCGCCTCGCTGGACGCTGAACAGCCAGGCCTGGAGGAGCACGTGGTGGGGCCACGCTGCCCCCAATGTGACCACGTCACGCTAGAGAACCTATCTGCGGGGCTGCTGCACCACCAGACCTTCGCTGCCTACGCGGCTGTGTATGGCGTGGCCCAGGCCCTTCACAACACACTGCGCTGCAATGCCTCGGGCTGCCCCAGGCGGGAGCCTGTGCGGCCCTGGCAGCTCCTAGAGAACATGTACAACGTGAGCTTCCGTGCTCGCGGCCTGGCACTGCAGTTCGACGCCAGCGGGAACGTGAACGTGGATTACGACCTGAAACTGTGGGTGTGGCAGGACCCGACGCCCGAGCTGCGCACCGTAGGCACCTTCAAGGGCCGCCTGGAGCTCTGGCGCTCTCAGATGTGCTGGCACACGCCGGGGAAGCAGCAGCCCGTGTCCCAGTGCTCCCGGCAGTGCAAGGAGGGCCAGGTGCGCCGCGTGAAGGGCTTCCACTCTTGCTGTTACGACTGCGTGGACTGCAAGGCGGGCAGTTATCAGCGCAACCCAGATGACCTCCTCTGCACCCAGTGTGACCAGGACCAGTGGTCCCCAGACCGGAGCACACGCTGCTTCGCCCGCAAGCCCATGTTCCTGGCATGGGGGGAGCCAGCTGTGCTGCTACTGCTCGCGCTGCTGGCTCTGGCGCTGGGCCTGGCGCTGGCAGCCCTGGGGCTCTTCCTCTGGCACTCGGACAGCCCGCTGGTTCAGGCCTCAGGTGGGCCACGGGCCTGCTTTGGCCTGGCCTGCCTGGGCCTGGTCTGCCTCAGTGTCCTCCTGTTCCCTGGCCAGCCAGGCCCTGCCAGCTGCCTGGCCCAGCAGCCACTGTTCCACCTCCCACTCACTGGCTGCCTGAGCACGCTTTTCCTGCAAGCGGCCGAGATATTTGTGGGGTCGGAGCTGCCACCAAGCTGGGCTGAGAAGATGCGTGGCCGCCTGCGGGGGCCCTGGGCCTGGCTGGTGGTGCTGCTTGCTATGCTGGCAGAAGCCGCATTGTGTGCCTGGTACCTGGTAGCCTTCCCGCCAGAGGTGGTGACGGACTGGCGGGTACTGCCCACAGAGGCGCTGGTGCACTGCCACGTGCACTCCTGGATCAGCTTCGGCCTGGTGCATGCCACTAACGCCATGCTGGCCTTCCTCTGCTTCCTGGGCACTTTCCTGGTGCAGAGCCGGCCAGGCCGCTACAATGGTGCCCGCGGCCTCACCTTTGCCATGCTGGCCTACTTCATCACCTGGATCTCCTTTGTGCCCCTCTTTGCCAATGTGCACGTGGCCTACCAGCCTGCCGTGCAGATGGGCACCATCCTCCTCTGTGCCCTGGGTATCCTAGCCACCTTCCACCTGCCCAAGTGCTACCTGCTGCTGCAGCGGCCGGAGCTCAACACCCCTGAGTTCTTCCTGGAAGACAATGCCAGAGCACAGGGCAGCAGTTGGGGGCAGGGGAGGGGAGAATCGGGGCAAAAACAAGTGACACCCGATCCAGTGACCTCACCGCAGTGA

**Cat Tas1r3 amino acid sequence**

MPGLALLGLTALLGLTALLDHGEGATSCLSQQLRMQGDYVLGGLFPLGSAEGTGLGDGLQPNATVCTRFSSLGLLWALAVKMAVEEINNGSALLPGLHLGYDLFDTCSEPMVAMKPSLVFMAKAGSCSIAAYCNYTQYQPRVLAVIGPHSSELALVTGKFFSFFLVPQVSYGASTDRLSNREIFPSFFRTVPSDQVQVAAMVELLQELGWNWVAAVGSDDEYGRQGLSLFSGLASARGICIAHEGLVPLPPGSLRLGALQGLLRQVNQSSVQVVVLFSSAHAARTLFSYSIRCKLSPKVWVASEAWLTSDLVMTLPGMPGVGTVLGFLQQGAPMPEFPSYVRTRLALAADPAFCASLDAEQPGLEEHVVGPRCPQCDHVTLENLSAGLLHHQTFAAYAAVYGVAQALHNTLRCNASGCPRREPVRPWQLLENMYNVSFRARGLALQFDASGNVNVDYDLKLWVWQDPTPELRTVGTFKGRLELWRSQMCWHTPGKQQPVSQCSRQCKEGQVRRVKGFHSCCYDCVDCKAGSYQRNPDDLLCTQCDQDQWSPDRSTRCFARKPMFLAWGEPAVLLLLALLALALGLALAALGLFLWHSDSPLVQASGGPRACFGLACLGLVCLSVLLFPGQPGPASCLAQQPLFHLPLTGCLSTLFLQAAEIFVGSELPPSWAEKMRGRLRGPWAWLVVLLAMLAEAALCAWYLVAFPPEVVTDWRVLPTEALVHCHVHSWISFGLVHATNAMLAFLCFLGTFLVQSRPGRYNGARGLTFAMLAYFITWISFVPLFANVHVAYQPAVQMGTILLCALGILATFHLPKCYLLLQRPELNTPEFFLEDNARAQGSSWGQGRGESGQKQVTPDPVTSPQ*

**Supplementary Figure 4.** Cat *Tas1r3* nucleic acid sequence and cat Tas1r3 amino acid sequence.

**Human *TAS1R1* nucleic acid sequence**

ATGCTGCTCTGCACGGCTCGCCTGGTCGGCCTGCAGCTTCTCATTTCCTGCTGCTGGGCCTTTGCCTGCCATAGCACGGAGTCTTCTCCTGACTTCACCCTCCCCGGAGATTACCTCCTGGCAGGCCTGTTCCCTCTCCATTCTGGCTGTCTGCAGGTGAGGCACAGACCCGAGGTGACCCTGTGTGACAGGTCTTGTAGCTTCAATGAGCATGGCTACCACCTCTTCCAGGCTATGCGGCTTGGGGTTGAGGAGATAAACAACTCCACGGCCCTGCTGCCCAACATCACCCTGGGGTACCAGCTGTATGATGTGTGTTCTGACTCTGCCAATGTGTATGCCACGCTGAGAGTGCTCTCCCTGCCAGGGCAACACCACATAGAGCTCCAAGGAGACCTTCTCCACTATTCCCCTACGGTGCTGGCAGTGATTGGGCCTGACAGCACCAACCGTGCTGCCACCACAGCCGCCCTGCTGAGCCCTTTCCTGGTGCCCATGATTAGCTATGCGGCCAGCAGCGAGACGCTCAGCGTGAAGCGGCAGTATCCCTCTTTCCTGCGCACCATCCCCAATGACAAGTACCAGGTGGAGACCATGGTGCTGCTGCTGCAGAAGTTCGGGTGGACCTGGATCTCTCTGGTTGGCAGCAGTGACGACTATGGGCAGCTAGGGGTGCAGGCACTGGAGAACCAGGCCACTGGTCAGGGGATCTGCATTGCTTTCAAGGACATCATGCCCTTCTCTGCCCAGGTGGGCGATGAGAGGATGCAGTGCCTCATGCGCCACCTGGCCCAGGCCGGGGCCACCGTCGTGGTTGTTTTTTCCAGCCGGCAGTTGGCCAGGGTGTTTTTCGAGTCCGTGGTGCTGACCAACCTGACTGGCAAGGTGTGGGTCGCCTCAGAAGCCTGGGCCCTCTCCAGGCACATCACTGGGGTGCCCGGGATCCAGCGCATTGGGATGGTGCTGGGCGTGGCCATCCAGAAGAGGGCTGTCCCTGGCCTGAAGGCGTTTGAAGAAGCCTATGCCCGGGCAGACAAGAAGGCCCCTAGGCCTTGCCACAAGGGCTCCTGGTGCAGCAGCAATCAGCTCTGCAGAGAATGCCAAGCTTTCATGGCACACACGATGCCCAAGCTCAAAGCCTTCTCCATGAGTTCTGCCTACAACGCATACCGGGCTGTGTATGCGGTGGCCCATGGCCTCCACCAGCTCCTGGGCTGTGCCTCTGGAGCTTGTTCCAGGGGCCGAGTCTACCCCTGGCAGCTTTTGGAGCAGATCCACAAGGTGCATTTCCTTCTACACAAGGACACTGTGGCGTTTAATGACAACAGAGATCCCCTCAGTAGCTATAACATAATTGCCTGGGACTGGAATGGACCCAAGTGGACCTTCACGGTCCTCGGTTCCTCCACATGGTCTCCAGTTCAGCTAAACATAAATGAGACCAAAATCCAGTGGCACGGAAAGGACAACCAGGTGCCTAAGTCTGTGTGTTCCAGCGACTGTCTTGAAGGGCACCAGCGAGTGGTTACGGGTTTCCATCACTGCTGCTTTGAGTGTGTGCCCTGTGGGGCTGGGACCTTCCTCAACAAGAGTGACCTCTACAGATGCCAGCCTTGTGGGAAAGAAGAGTGGGCACCTGAGGGAAGCCAGACCTGCTTCCCGCGCACTGTGGTGTTTTTGGCTTTGCGTGAGCACACCTCTTGGGTGCTGCTGGCAGCTAACACGCTGCTGCTGCTGCTGCTGCTTGGGACTGCTGGCCTGTTTGCCTGGCACCTAGACACCCCTGTGGTGAGGTCAGCAGGGGGCCGCCTGTGCTTTCTTATGCTGGGCTCCCTGGCAGCAGGTAGTGGCAGCCTCTATGGCTTCTTTGGGGAACCCACAAGGCCTGCGTGCTTGCTACGCCAGGCCCTCTTTGCCCTTGGTTTCACCATCTTCCTGTCCTGCCTGACAGTTCGCTCATTCCAACTAATCATCATCTTCAAGTTTTCCACCAAGGTACCTACATTCTACCACGCCTGGGTCCAAAACCACGGTGCTGGCCTGTTTGTGATGATCAGCTCAGCGGCCCAGCTGCTTATCTGTCTAACTTGGCTGGTGGTGTGGACCCCACTGCCTGCTAGGGAATACCAGCGCTTCCCCCATCTGGTGATGCTTGAGTGCACAGAGACCAACTCCCTGGGCTTCATACTGGCCTTCCTCTACAATGGCCTCCTCTCCATCAGTGCCTTTGCCTGCAGCTACCTGGGTAAGGACTTGCCAGAGAACTACAACGAGGCCAAATGTGTCACCTTCAGCCTGCTCTTCAACTTCGTGTCCTGGATCGCCTTCTTCACCACGGCCAGCGTCTACGACGGCAAGTACCTGCCTGCGGCCAACATGATGGCTGGGCTGAGCAGCCTGAGCAGCGGCTTCGGTGGGTATTTTCTGCCTAAGTGCTACGTGATCCTCTGCCGCCCAGACCTCAACAGCACAGAGCACTTCCAGGCCTCCATTCAGGACTACACGAGGCGCTGCGGCTCCACCTGA

**Human TAS1R1 amino acid sequence**

MLLCTARLVGLQLLISCCWAFACHSTESSPDFTLPGDYLLAGLFPLHSGCLQVRHRPEVTLCDRSCSFNEHGYHLFQAMRLGVEEINNSTALLPNITLGYQLYDVCSDSANVYATLRVLSLPGQHHIELQGDLLHYSPTVLAVIGPDSTNRAATTAALLSPFLVPMISYAASSETLSVKRQYPSFLRTIPNDKYQVETMVLLLQKFGWTWISLVGSSDDYGQLGVQALENQATGQGICIAFKDIMPFSAQVGDERMQCLMRHLAQAGATVVVVFSSRQLARVFFESVVLTNLTGKVWVASEAWALSRHITGVPGIQRIGMVLGVAIQKRAVPGLKAFEEAYARADKKAPRPCHKGSWCSSNQLCRECQAFMAHTMPKLKAFSMSSAYNAYRAVYAVAHGLHQLLGCASGACSRGRVYPWQLLEQIHKVHFLLHKDTVAFNDNRDPLSSYNIIAWDWNGPKWTFTVLGSSTWSPVQLNINETKIQWHGKDNQVPKSVCSSDCLEGHQRVVTGFHHCCFECVPCGAGTFLNKSDLYRCQPCGKEEWAPEGSQTCFPRTVVFLALREHTSWVLLAANTLLLLLLLGTAGLFAWHLDTPVVRSAGGRLCFLMLGSLAAGSGSLYGFFGEPTRPACLLRQALFALGFTIFLSCLTVRSFQLIIIFKFSTKVPTFYHAWVQNHGAGLFVMISSAAQLLICLTWLVVWTPLPAREYQRFPHLVMLECTETNSLGFILAFLYNGLLSISAFACSYLGKDLPENYNEAKCVTFSLLFNFVSWIAFFTTASVYDGKYLPAANMMAGLSSLSSGFGGYFLPKCYVILCRPDLNSTEHFQASIQDYTRRCGST*

**Supplementary Figure 5.** Human *TAS1R1* nucleic acid sequence and human TAS1R1 amino acid sequence.

***In vitro* concentration-response curves for non-active L-amino acids and taurine.**

**Supplementary Figure 6.** *In vitro* concentration-response curves for L-amino acids and taurine that were non-active with the cat Tas1r1-Tas1r3 either with or without 0.2 mM IMP. Change in fluorescence (∆F/ F) is shown on the y-axis and amino acid concentration (mM) is shown on the x-axis. Blue broken line is cat Tas1r1-Tas1r3 without IMP, blue continuous line is cat Tas1r1-Tas1r3 with 0.2 mM IMP, black broken line is mock without IMP, and black continuous line is mock with 0.2 mM IMP.

**Material and Methods for *in vivo* response of cats using 1-hr exposure period.**

All taste choice tests with 1-hr exposure period were run as outlined in the Materials and Methods, with the following differences. The minimum panel size was n = 24 and the maximum panel size was n = 25. The cats had an age of 1.43 - 9.11 years, with the proportion of males varying between 0.71 - 0.80 (accordingly, the proportion of females varied between 0.20 - 0.29) during the studies. The cats were water-restricted for 1-hr prior to the 1-hr water exposure period, with their first meal given in the last 20-min of the water restriction. 50 mL of each test solution was offered to the cats in round water bowls with a maximum capacity 150 mL (Flyte So Fancy, UK), which were held in bespoke bowl holders (TECHNIK – A Division of Syspal Ltd., UK).

***In vivo* response of cats to nucleotides using 1-hr exposure period.**

A


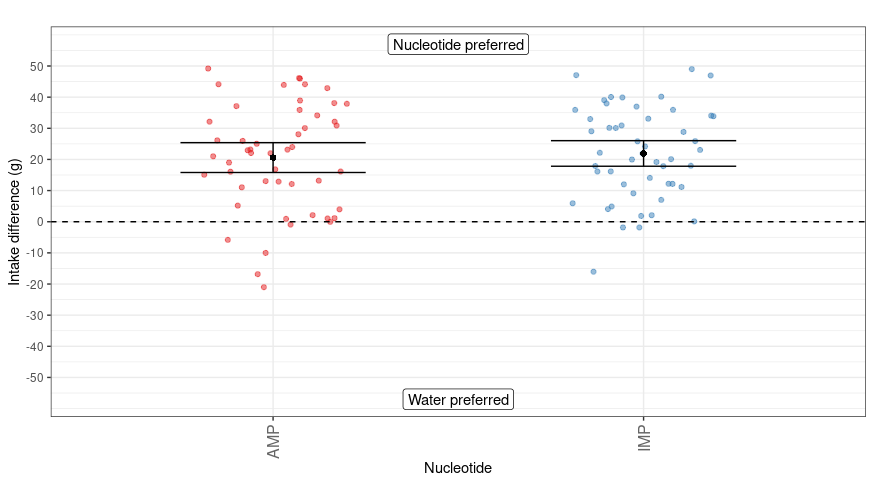


B

| Nucleotide: | Intake diff. (g): | 95% CI (g): | *p*-value: | n: |
| --- | --- | --- | --- | --- |
| AMP | 20.6 | (15.8, 25.4) | < 0.001 | 25 |
| IMP | 21.9 | (17.8, 26.0) | < 0.001 | 25 |

**Supplementary Figure 7.** A). *In vivo* response of cats to 2 nucleotides using a Water Panel with 1-hr exposure period. The difference in intake (g) is shown on the y-axis and the nucleotides tested are shown on the x-axis. Means are shown with 95% confidence intervals. The nucleotides are ordered from left to right, from lowest to highest difference in intake (g). All nucleotides were tested at 5 mM. B). Nucleotide intake difference values (g) ordered from lowest to highest, with 95% confidence intervals (CI), p-values, and n values.

***In vivo* response of cats to L-amino acids using 1-hr exposure period.**

A


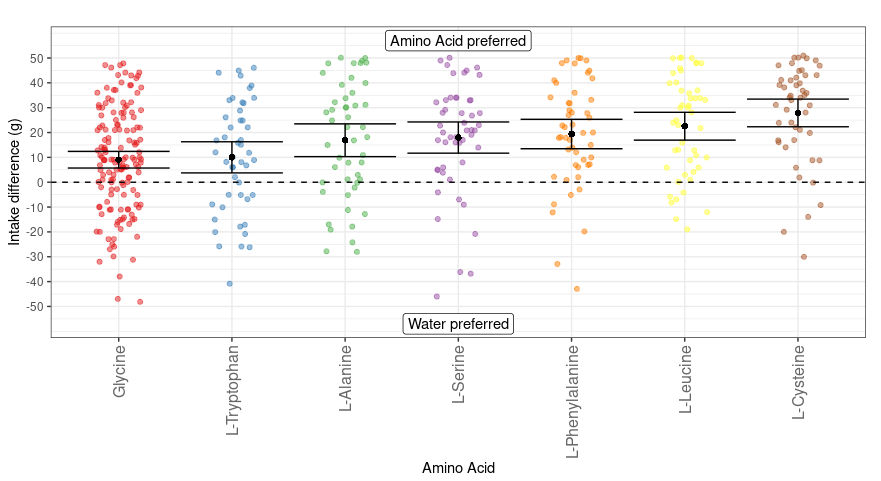


B

| Amino acid: | Intake diff. (g): | 95% CI (g): | *p*-value: | n: |
| --- | --- | --- | --- | --- |
| Glycine | 9.1 | (5.7, 12.4) | < 0.001 | 75 (25) |
| L-Tryptophan | 10.0 | (3.8, 16.3) | < 0.001 | 24 |
| L-Alanine | 16.9 | (10.3, 23.5) | < 0.001 | 24 |
| L-Serine | 18.0 | (11.7, 24.3) | < 0.001 | 25 |
| L-Phenylalanine | 19.4 | (13.5, 25.3) | < 0.001 | 25 |
| L-Leucine | 22.5 | (16.9, 28.1) | < 0.001 | 24 |
| L-Cysteine | 27.9 | (22.3, 33.5) | < 0.001 | 24 |

**Supplementary Figure 8.** A). *In vivo* response of cats to 7 L-amino acids using a Water Panel with 1-hr exposure period. The difference in intake (g) is shown on the y-axis and the amino acids tested are shown on the x-axis. Means are shown with 95% confidence intervals. The amino acids are ordered from left to right, from lowest to highest difference in intake (g). All amino acids were tested at 25 mM. B). Amino acid intake difference values (g) ordered from lowest to highest, with 95% confidence intervals (CI), p-values, and n values (where multiple tests were run, the number of unique cats is shown in brackets).
